# Differential downstream signaling in microglia lacking Alzheimer’s-related TREM2 or its adaptor TYROBP/DAP12

**DOI:** 10.1186/s44477-025-00012-x

**Published:** 2026-01-19

**Authors:** Gabriela E. Farias Quipildor, Ramona Belfiore, Khaled Althobaiti, Zahra Najarzadeh, Charles Glabe, Benjamin P. Readhead, Sam Gandy, Stephen R. J. Salton, Michelle E. Ehrlich

**Affiliations:** 1 Department of Neurology, Icahn School of Medicine at Mount Sinai, New York, NY 10029 USA; 2 Nash Family Department of Neuroscience, Icahn School of Medicine at Mount Sinai, New York, NY 10029 USA; 3 Friedman Brain Institute, Icahn School of Medicine at Mount Sinai, New York, NY 10029 USA; 4 Department of Molecular Biology and Biochemistry, University of California, Irvine, CA 92697 USA; 5 ASU-Banner Neurodegenerative Disease Research Center, Arizona State University, Tempe, AZ USA; 6 Department of Psychiatry and Alzheimer’s Disease Research Center, Icahn School of Medicine at Mount Sinai, New York, NY 10029 USA; 7 James J Peters VA Medical Center, Bronx, NY 10468 USA; 8 Department of Pediatrics, Icahn School of Medicine at Mount Sinai, New York, NY 10029 USA; 9 Department of Genetics and Genomic Sciences, Icahn School of Medicine at Mount Sinai, New York, NY 10029 USA; 10 Present Address: Duchenne Regulatory Science Consortium, Critical Path Institute, 1840 East River Road, Suite 100, Tucson, AZ 85718 USA; 11 Department of Geriatrics and Palliative Medicine, Icahn School of Medicine at Mount Sinai, New York, NY 10029 USA

**Keywords:** TREM2, TYROBP, DAP12, Microglia, Signal transduction

## Abstract

Microglia, the primary immune cell in the brain, have multiple activation phenotypes involved in broad functions within the brain, playing roles in neurotoxicity/neuroprotection, release of inflammatory and anti-inflammatory cytokines, and in cell survival, proliferation, and phagocytosis. TREM2 and TYROBP form a transmembrane complex in microglia that modulates intracellular signaling networks, and these proteins are essential regulators of the transition from homeostatic to activated microglia. Recent findings support a TREM2-independent molecular signature that is involved in the early transition of homeostatic to disease-associated microglia (DAM), with the next sequential step of DAM activation from stage 1 to stage 2 being TREM2-dependent. However, the underlying mechanisms determining how TREM2 or TYROBP regulate these downstream phenotypes are largely unknown. We isolated primary microglia from C57BL/6 wild-type (WT) controls, *Trem2* knock-out (KO), and *Tyrobp* KO mice at post-natal day 0–3. Cells were treated with Alzheimer’s disease (AD)-relevant stimuli, such as amyloid beta (Aβ) oligomers or fibrils, or ‘neuroinflammatory-like’ stimuli, such as lipopolysaccharide (LPS). We explored protein and gene expression in the presence or absence of inhibitors of the TREM2/TYROBP downstream signaling pathway. We also performed a high-throughput Olink proteomic analysis of conditioned media from WT, *Trem2* KO, and *Tyrobp* KO stimulated with either LPS or Aβ oligomers or fibrils. Our results show that the absence of either TREM2 or TYROBP is associated with increased basal levels of phosphorylated ERK in primary microglia compared to WT controls. In addition, *Trem2* KO and *Tyrobp* KO cells show a less ramified cell morphology at baseline, as compared to WT microglia. Moreover, stimulating primary microglia with either Aβ oligomers or LPS leads to differential protein and gene expression in cells lacking TREM2 or TYROBP. The dysregulated downstream signal transduction and morphology in the absence of TREM2 or TYROBP suggest their essential roles not only in microglial homeostasis but also in their activation in response to different stimuli.

**Graphical abstract:**

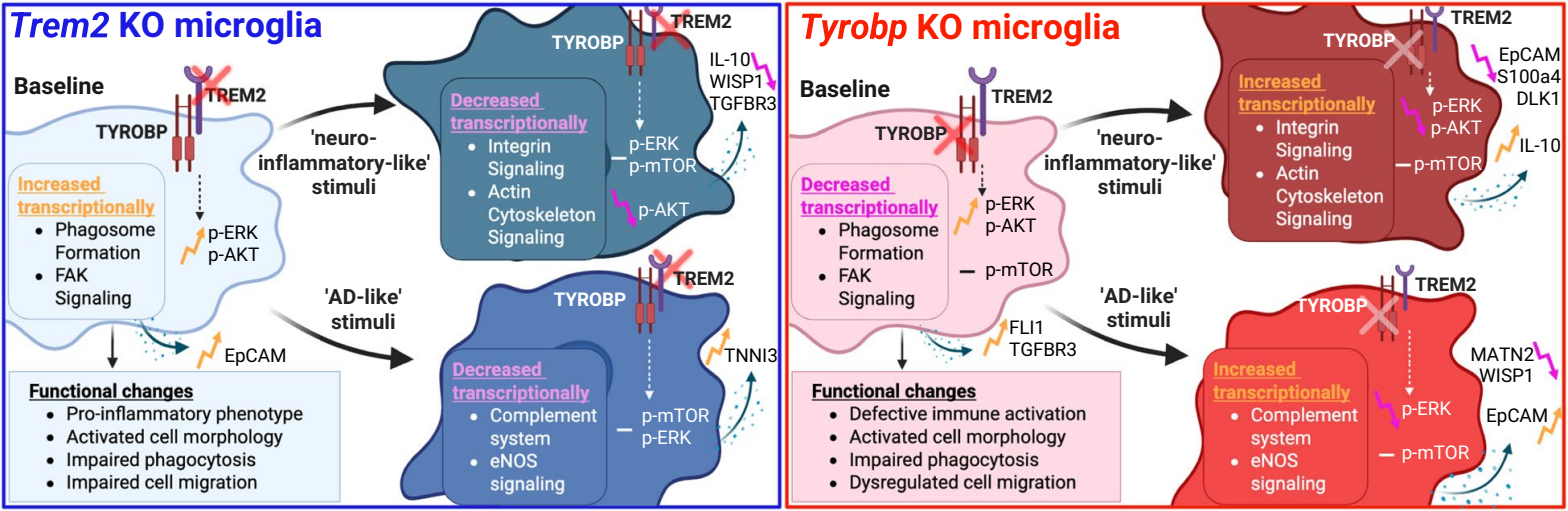

**Supplementary Information:**

The online version contains supplementary material available at 10.1186/s44477-025-00012-x.

## Background

Alzheimer’s disease (AD) is a progressive neurodegenerative disease characterized by a decline in cognitive function, memory loss, and changes in behavior and personality. AD neuropathology has been primarily defined by a widespread extracellular accumulation of amyloid beta (Aβ) peptides and an intracellular buildup of hyperphosphorylated tau-containing neurofibrillary tangles [1]. Aβ plaque deposition usually is thought of as an imbalance between the production and clearance of these peptides [2, 3]. Aβ plaques have a dense core of compacted fibrillar Aβ surrounded by soluble Aβ oligomers, which are hypothesized to be the most neurotoxic forms of Aβ peptides [2, 4].

In addition to protein aggregates, patients with mild cognitive impairment (which can be an early harbinger of AD) have evidence of Aβ deposition and activated microglia in overlapping areas, as shown by positron emission tomography [5]. AD brains also show microglial activation, a hallmark of neuroinflammation [6]. Specific microglial cell surface receptors, including TREM2 and CD33, are activated in pathological states, including AD, and mutations in the genes encoding these microglial proteins increase the risk of susceptibility to developing the disease [7, 8]. TREM2 is a transmembrane glycoprotein with a short intracellular domain that contains no identified signaling motifs, and an extracellular domain that binds to lipopolysaccharide (LPS) [9], high-density and low-density lipoproteins [10, 11], several apolipoproteins [12], Aβ peptides [13, 14], and cellular debris [15].

To initiate signal transduction, TREM2 associates with TYROBP (also known as DAP12), which results in phosphorylation of the TYROBP immuno-tyrosine activation motif (ITAM) by Src kinases, creating a docking site for Syk (spleen tyrosine kinase). The TREM2/TYROBP axis activates signaling cascades that trigger mitogen-activated protein kinase (MAPK), phosphatidylinositol-3 kinase (PI3K), phospholipase C gamma (PLCγ), and the VAV protein family, to modulate microglial survival/motility/proliferation [16, 17], phagocytosis [15], and inflammatory responses [18].

A subpopulation of microglia, known as disease-associated microglia (DAM), clusters around Aβ plaques and becomes activated by a two-step mechanism: a) the first step from homeostatic to stage 1 DAM is TREM2-independent, while b) the transition from stage 1 DAM to stage 2 DAM is TREM2-dependent [19–21]. Our group has previously shown a TREM2-independent upregulation of *Tyrobp* and *Apoe* in two different mouse models of Aβ amyloidosis, suggesting that TYROBP-APOE signaling could be involved in stage 1 of DAM activation [22]. In addition, other reactive microglial populations have been characterized in other neurodegeneration models, including IFN-responsive microglia and microglia with a proliferative and metabolically active signature [23–25].

Another mechanism by which the TREM2/TYROBP axis generates conflicting signals is by low avidity binding of ligands to TREM2. When only partial phosphorylation of the ITAM motif in TYROBP occurs, SHP-1 phosphatase is activated, which subsequently dephosphorylates molecules downstream from Syk, and leads to inhibition of cellular activation [26]. This suggests a significant complexity in the TREM2/TYROBP axis that requires further investigation to understand microglial activation. Although genetic mutations of TREM2 and TYROBP and their effects on cell survival/motility/proliferation, phagocytosis, and inflammation in microglia have been characterized in both mice and humans, the underlying mechanisms of how these proteins affect downstream function remain incompletely understood.

Therefore, in this study, we evaluated the microglial signaling pathways that are regulated in response to a “neuroinflammatory-like” state, by stimulating primary microglia with LPS, or in response to AD-like stimuli, by treating primary microglia with either OC-type Aβ fibrils alone or with a mixture of A11- and OC-type Aβ proteoforms [27]. We hypothesized that ablation of either TREM2 or TYROBP in microglia would lead to differentially dysregulated cellular activation in response to disease-relevant stimuli. To our knowledge, this is the first study that directly contrasts the signaling pathways utilized by TREM2 and TYROBP to regulate microglial homeostatic and activated functions downstream from TREM2 or TYROBP, in an isolated primary microglial setting.

## Methods

### Study design

Primary microglia were isolated from wild-type (WT), *Trem2* knockout (*Trem2* KO), and *Tyrobp* knockout (*Tyrobp* KO) mice. Primary cells were grown in Dulbecco’s modified Eagle’s medium (DMEM, Gibco, Cat. # 10566024) supplemented with 10% Heat Inactivated Fetal Bovine Serum (ThermoScientific, Cat. # 10438026), 1% Penicillin/Streptomycin (ThermoScientific, Cat. # 15140122), and 1% non-essential amino acids. Cells were seeded in the afternoon, allowed to attach, grown overnight, and used within the next 16–20 h. First, cells were pre-treated for 4 h with either: serum-free unsupplemented growth media, Syk inhibitor (5 µM BAY61-3606); PP1/PP2A phosphatase inhibitor (40 nM okadaic acid); or Toll-like receptor 4 (TLR4) inhibitor (5 µMTAK-242), all of which were diluted in serum-free unsupplemented growth media. After pharmacologic inhibition, cells were stimulated for 0, 5, 15, 30 min, or 24 h with a final concentration of 1µM of LPS or with a final concentration of 0.20 µM of Aβ oligomers for 24 h in serum-free unsupplemented growth media. After these treatments, conditioned media were collected, and protein lysates or total RNA were prepared. The experimental design is depicted in Supplementary Fig. 1.

### Animals

C57BL/6 mice were used as WT controls. *Trem2* KO mice were constructed by removing exon 1 and exon 2 by targeted homologous recombination, as reported [22]. *Tyrobp* KO mice were obtained from Taconic/Merck Laboratory. All animal lines were bred in-house and maintained homozygous and in a 12-h/12-h light/dark cycle. All experimental procedures were conducted in accordance with NIH Guidelines for Animal Research, and all protocols were approved by the Institutional Animal Care and Use Committee of the Icahn School of Medicine at Mount Sinai.

### Primary microglia isolation

Primary microglia were isolated from newborn WT, *Trem2* KO, and *Tyrobp* KO mice and maintained at 37 °C, 5% CO_2_ in a humidified incubator. In brief, postnatal day 0–3 pups were decapitated, and meninges were removed. Hippocampi and cortices were collected and mechanically homogenized in Hibernate A minus phenol red medium (Transnetyx Tissue by BrainBits, Cat. # SKU HAPR500). CD11b + magnetic beads were used to perform a magnetic cell separation via MACS technology (Miltenyi Biotec), as described [28]. Cells were then collected, counted, and seeded at a density of 100,000 cells per well in 24-well plates for downstream analyses.

### Preparation of OC-type Aβ42 fibril

To prepare the stock solution, 0.3 mg of Aβ42 peptide was dissolved in 50 µL of 50 mM NaOH. The solution was then sonicated in a bath ultrasonic for two cycles (30 s ON, 30 s OFF). Then, the monomeric peptide was centrifuged at 14,000 × g to remove any undissolved peptide or aggregates. The supernatant was transferred to a new vial and diluted to a final concentration of 100 µM in 20 mM phosphate buffer (pH 7.5). The peptide solution was then incubated at 37 °C for three days in a 96-well plate. In three separate wells, Thioflavin T (ThT) was added at a final concentration of 30 µM to monitor fibrillation. However, the fibrils used in these experiments were obtained from wells that did not contain ThT.

### Preparation of Aβ42 oligomer and fibril mixture

To prepare the A11/OC positive mixture, 1 mg of Aβ42 peptide was dissolved in 300 µL of 100% HFIP (1,1,1,3,3,3-hexafluoro-2-propanol) and sonicated in a water bath for 5 min. The HFIP was then evaporated under a stream of N₂ gas, leaving behind a thin film of monomerized Aβ42. The dried film of Aβ42 was dissolved in 45 µL of 10% (v/v) NH₄OH and further diluted with 885 µL of ice-cold PBS (pH 12.0, adjusted with NaOH) to achieve a final concentration of 250 µM Aβ42. The solution was then centrifuged at 14,000 × g for 10 min at 4 °C. The resulting supernatant was sonicated in a water bath for 10 min and then ultra-centrifuged at 70,000 rpm (TLA110) for 1 h at 4 °C. The supernatant was transferred to a new vial following centrifugation, and 80 µL of 10 N NaOH was added. The sample was then diluted fivefold in 10% (v/v) NH₄OH to reach a final concentration of 50 µM Aβ42. Then, the solution was sonicated on ice for 10 min, aliquoted, and stored at −80 °C until use. Supplementary Fig. 2 shows electron microscopy images of Aβ40 and Aβ42 oligomers and fibrils, as previously reported [29], illustrating the morphology of the prepared aggregates prior to cellular treatment.

### Wound-healing assays

For cell migration assays, a plastic pipette tip was used to scratch the primary cell culture surface, and then the medium was changed to either serum-free unsupplemented media alone, or mixed with LPS, A11/OC, or OC-only preparation. The plate was placed in a Cytation 5 system (BioTek, USA) at 37 °C and 5% CO_2_, and images were taken at 0 and 12 h. The number of invading cells was counted using CellPose (machine learning based segmentation) [30]. The training stage was performed using the default training parameters settings with the “cyto3” model in CellPose. Cell density in one frame was calculated from the cell masks obtained from each frame.

### Phagocytosis assays

For phagocytosis assays, green fluorescent latex beads (Sigma #L1030) were added to primary WT and KO cell cultures either in serum-free unsupplemented media alone, or mixed with LPS, A11/OC, or OC-only preparations. In brief, beads were pre-opsonized for 1 h at 37 °C in heat inactivated FBS (Gibco) before use [31]. The final concentrations for beads and FBS in DMEM were 0.01% (v/v) and 0.05% (v/v), respectively. The plate was placed in a Cytation 5 system (BioTek, USA) at 37 °C and 5% CO_2_, and images were taken for 24 h. Beads within primary microglia were quantified in an automated fashion using BioTek Gen5 Software (Agilent) by creating an object mask for primary microglia with a secondary mask to count the subpopulation of cells that contained green fluorescent beads.

### Protein extraction and Western blotting

Proteins from primary microglial cells were extracted using 1X SDS lysis buffer (from 10X Western Lysis Buffer, Phosphosolutions) with 1X protease inhibitor cocktail (from 100X Halt Protease Inhibitor Cocktail, ThermoScientific) in order to completely solubilize membrane proteins and difficult-to-solubilize proteins involved in cell signaling [32]. Lysed protein was separated by electrophoresis in precast 4–12% Bis–Tris gels (Bio-Rad) and transferred to nitrocellulose membranes (Bio-Rad). Membranes were immunoblotted for TREM2/TYROBP downstream signaling targets, including phospho-Syk (Tyr^352^ and Tyr^525/526^), phospho-p44/p42 MAPK (ERK1/2) (Thr^202^/Tyr^204^), phospho-AKT (Ser^473^ and Thr^308^), phospho-mTOR (Ser^2448^), and their total counterparts – Supplementary Table 1 shows catalog numbers for the antibodies used in this study. ECL western blot substrate was used to develop immunoreactive proteins, Chemidoc (Bio-Rad) was used to image the blots, and ImageLab software (Bio-Rad) was used to quantify protein densitometry (See Supplementary File 3 for unedited blots).

### RNA extraction and gene expression assays

Total RNA from primary microglia was isolated using Qiazol and the miRNeasy Micro Kit (Qiagen), per manufacturer’s guidelines. First-strand complementary DNA (cDNA) was synthesized with random primers and total RNA as a template using the High-Capacity RNA-to-cDNA Kit (ThermoFisher Scientific). Real-time PCR reactions were prepared using the All-in-One qPCR Mix (GeneCopoeia).

### Immunofluorescence imaging

For primary microglia immunostaining, cells were fixed in ice-cold 4% paraformaldehyde in PBS, permeabilized in 0.3% Triton X-100 in 0.1 M PBS for 10 min, incubated in 0.1% Triton X-100 in 0.1 M PBS with 10% normal goat serum for 90 min at room temperature, and then incubated overnight at 4 °C in primary antibodies diluted (Supplementary Table 1) in 10% normal goat serum and 0.1% Triton X-100. After cells were washed with 0.1 M PBS, they were incubated with secondary Alexa-Fluor antibodies (Supplementary Table 1) in 0.1% BSA for 4 h at room temperature and mounted with DAPI. Imaging was conducted using the Biotek Cytation 5 cell imaging multimode reader (Agilent, CA) at a 10X magnification and analyzed using the Gen5 software version 3.12.08 (Agilent, CA). Circularity index was calculated by measuring the area and the perimeter of a cell (4π[area]/[perimeter]^2^) from a secondary mask.

### RNA sequencing and analysis

RNA samples from primary microglia were sequenced for transcriptomic profiling using the Illumina NovaSeq PE150 platform by Novogene. RNA quality of each sample was assessed by Novogene and cDNA libraries (150 bp, paired end reads) were sequenced using an ultra-low input protocol due to low RNA concentrations and quantity. RNA sequencing was performed on two replicates from three independent experiments per group. RNA reads were aligned to the *Mus Musculus* Ensembl Reference genome (GRCm38.77) using STAR aligner software [33] (Version 2.5.2b), and accepted mapped reads were summarized to gene level counts using the –quantMode option. Gene count filtering, normalization, and differential expression analysis were performed in R using the Limma package [34, 35]. Mouse gene identifiers were converted to available human orthologs using the Mouse Genome Database [36]. Differentially expressed genes (DEGs) were defined as showing a log_2_ fold change > 1.5, and FDR < 0.05, and considered statistically significant for pathway analysis. Ingenuity Pathway Analysis software (IPA, Qiagen) was used to analyze the most significant canonical pathways in our dataset as previously described [37]. The genes from the datasets associated with canonical pathways in the Ingenuity Pathways Knowledge Base were considered for further literary analysis.

### Olink proteomics

Conditioned media were collected from primary cell cultures untreated or treated with either LPS, A11/OC mix, or OC-only preparations for 24 h. Samples were submitted to the Mount Sinai Human Immune Monitor Center to be analyzed using the Olink Target 96 mouse exploratory proteomics platform, which measured 92 biomarkers simultaneously. The panel uses proximity extension assay (PEA) technology, which involves binding oligonucleotide-labeled antibody probe pairs to their respective target protein when in close proximity. A real-time PCR reaction is used to amplify the signal from DNA-labeled antibody pairs and quantify the relative concentration of protein in the sample. Protein concentrations are normalized from Ct values to Normalized Protein eXpression (NPX) arbitrary units on a log_2_ transformed distribution, where higher NPX values correspond to higher protein concentrations.

### Statistical methods

Cross-sectional data were analyzed using a two-way analysis of variance (ANOVA) to assess the main effects of genotype and treatment, as well as their interaction. When a significant effect was observed, post hoc comparisons were performed using Tukey’s multiple comparisons tests. All statistical analyses were conducted using GraphPad Prism version 10.6.1 for Mac (GraphPad Software, San Diego, CA). All values are presented as means ± standard error of the mean (SEM), and differences were considered statistically significant at *p* ≤ 0.05.

## Results

### *Trem2* KO and *Tyrobp* KO microglia show similar disruptions in cell morphology, phagocytosis, and migration at baseline, but present functional differences upon LPS or Aβ stimulation

**Fig. 1:**
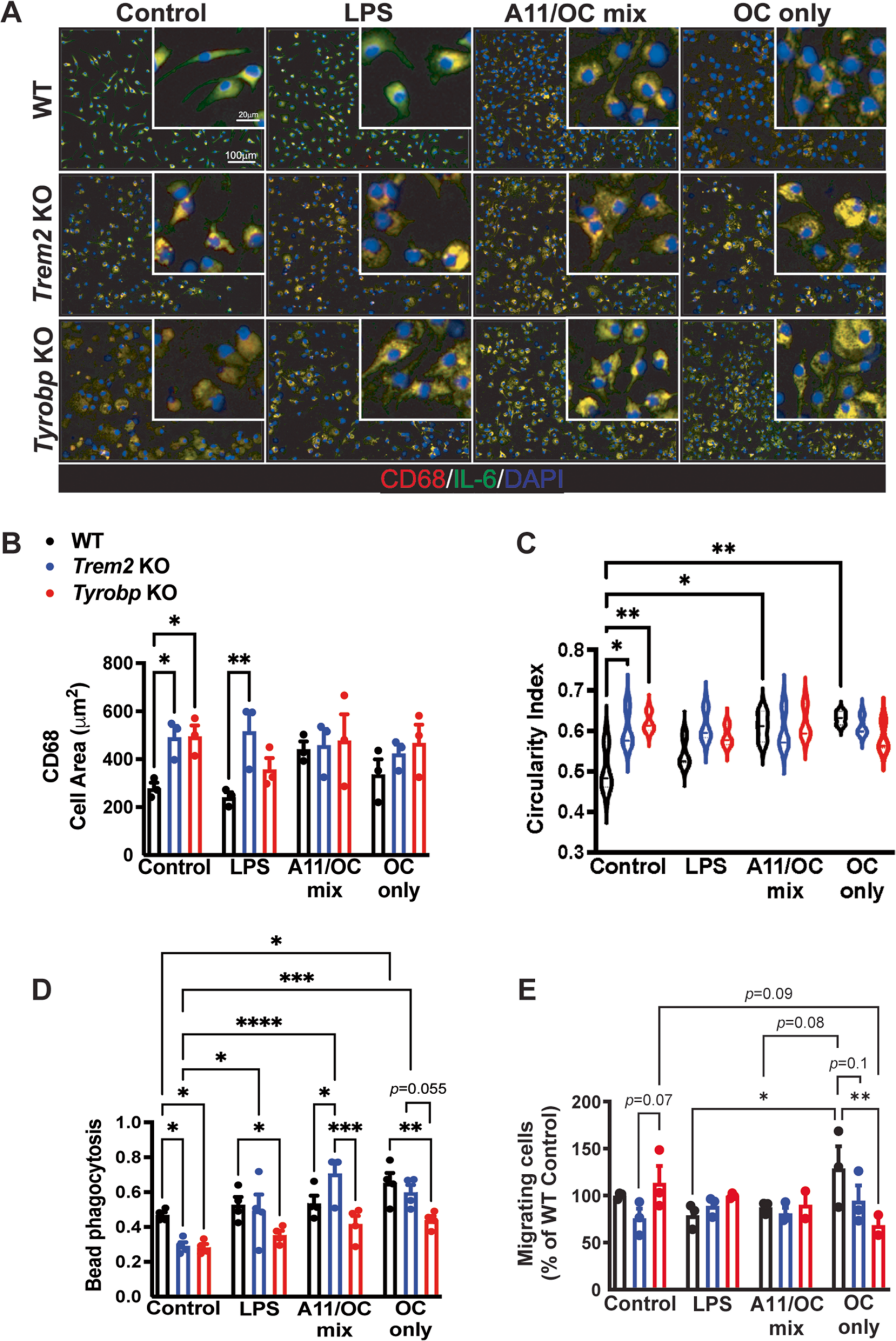
Functional disruptions of *Trem2* KO and *Tyrobp* KO microglia at baseline, and upon stimulation. **A)** Representative images of CD68 (red), IL-6 (green) and DAPI (blue) staining from WT, *Trem2* KO, and *Tyrobp* KO microglia at baseline and after a 24-h stimulation with either LPS, A11/OC mix or OC-only preparations. Cells co-stained for CD68 and IL-6 appear yellow. **B)** Plot shows individual cells analyzed for cell area quantification (based on CD68 staining). **C)** Manual morphological analysis was performed using the Gen5 software (BioTek) by measuring the area and the perimeter of a cell. The circularity index was calculated for all groups and is represented in plot **C**. **D)** Green-fluorescent bead phagocytosis quantification from WT, *Trem2* KO, and *Tyrobp* KO is shown at baseline and after 24-h treatment with either LPS, A11/OC mix or OC-only preparations. Green # signs and *p-*values represent comparisons between each stimulation group to its baseline control.** E)** Migrating cell quantification from WT, *Trem2* KO, and *Tyrobp* KO at baseline and after 12-h treatment with either LPS, A11/OC mix or OC-only preparations. WT controls were set to 100% and the rest of the groups show the percent change compared to WT controls. **A-E)** *N* = 3 independent experiments. Bars represent means ± SEM. Black bars indicate WT microglia, blue bars represent *Trem2* KO microglia, and red bars represent *Tyrobp* KO microglia. In the violin plots, the horizontal line within each distribution denotes the median value. Data were analyzed using a two-way ANOVA followed by Tukey’s multiple comparisons test. *p < 0.05

Microglial activation in response to pathology in the brain has been characterized as a continuum of changes in cell morphology, phagocytosis, and migration [38]. Therefore, we assessed overall activation in WT, *Trem2* KO, and *Tyrobp* KO mouse microglia at baseline as well as after LPS stimulation, or treatment with either a preparation of OC-type Aβ fibrils or with a mixture of A11- and OC-type Aβ proteoforms (Fig. 1).

WT primary microglia, as reported [39, 40], showed an elongated uniformly ramified morphology with short processes and a small cell body at baseline (Fig. 1A). Observationally, the processes of WT primary microglia decrease in length after LPS stimulation while their cell area slightly increases after exposure to the mixture of prefibrillar Aβ oligomers and fibrils, or isolated fibrils (Fig. 1A, B). In contrast, both *Trem2* KO and *Tyrobp* KO show a different morphology to WT microglia with shorter processes and bigger cell bodies at baseline (Fig. 1A, B). After LPS stimulation, *Tyrobp* KO microglia showed a decrease in cell area quantified by CD68 staining, while no change was observed in *Trem2* KO microglia (Fig. 1A, B). Similar results were confirmed when analyzing morphology by manually calculating the circularity index (Fig. 1C). After Aβ stimulation, the circularity index in WT microglia increased, however, the cell area or circularity index of neither *Trem2* KO nor *Tyrobp* KO microglia changed in response to a 24-h treatment (when compared to baseline of each respective genotype) (Fig. 1A-C). This would suggest not only that *Trem2* KO and *Tyrobp* KO microglia have an activated microglial state at baseline, compared to WT, but also that *Tyrobp* KO microglia are more responsive than *Trem2* KO microglia to an LPS stimulus.

To assess phagocytosis, the uptake of green, fluorescent beads was measured at baseline and after LPS or Aβ stimulation of WT, *Trem2* KO and *Tyrobp* KO microglia (Fig. 1D). After 24 h, both *Trem2* KO and *Tyrobp* KO microglia had a decrease in bead phagocytosis, compared to WT control microglia, despite the absence of stimulation (Fig. 1D). Upon LPS or Aβ stimulation, bead phagocytosis increased in *Trem2* KO compared their baseline, while *Tyrobp* KO microglia showed no stimulation-induced increase in phagocytosis (Fig. 1D). Therefore, while both genotypes have impaired phagocytic capacity relative to WT at baseline, *Trem2* KO microglia have greater responsiveness to activation stimuli than *Tyrobp* KO microglia, suggesting a more pronounced phagocytic defect in the absence of TYROBP.

Next, we assessed cell migration using the scratch-wound assay at baseline and after LPS or Aβ stimulation (Fig. 1E and Supplementary Fig. 3). There were no differences in cell migration between *Trem2* KO and *Tyrobp* KO compared to WT control microglia, although *Trem2* KO microglia trended toward decreased migration compared to *Tyrobp* KO under baseline conditions (Fig. 1E). However, upon different stimulations, WT and *Trem2* KO microglial motility did not significantly change while *Tyrobp* KO microglial migration decreased after treatment with isolated Aβ fibrils (Fig. 1E).

In summary, the lack of TREM2 or TYROBP provoked similar changes in cell morphology and bead phagocytosis, while there were differences in cell migration at baseline. LPS stimulation of microglia lacking TYROBP resulted in more significant changes in cell morphology than in *Trem2* KO microglia; while in *Trem2* KO microglia, LPS increased bead phagocytosis to a greater extent than in *Tyrobp* KO microglia and had no effect on cell migration. Isolated Aβ fibrils increased bead phagocytosis in *Trem2* KO microglia considerably more than in *Tyrobp* KO microglia, decreased cell migration in Tyrobp KO, and had no impact on cell morphology, all of which is summarized in Table 1.

**Table 1:** Summary of functional assays in Fig. 1

Summary	WT	*Trem2* KO	*Tyrobp* KO
Morphology at baseline	Ramified	Amoeboid	Amoeboid
Morphology after stimulation	Amoeboid	No change from baseline	No change from baseline
Phagocytosis at baseline	⎯	↓	↓
Phagocytosis after stimulation	↑↑	↑↑	No change from baseline
Migration at baseline	⎯	↓	↑
Migration after stimulation	No change in LPS ↑ in Aβ	No change from baseline	↓ in Aβ

Baseline, up and down arrows indicate comparison of KO to WT at baseline. After stimulation, up and down arrows indicate comparison to baseline measurements for each respective genotype. Dash line indicates no comparison performed

### LPS stimulation increases ERK and mTOR phosphorylation in a time-dependent manner in WT primary microglia, but not in *Trem2* KO or *Tyrobp* KO microglia

**Fig. 2:**
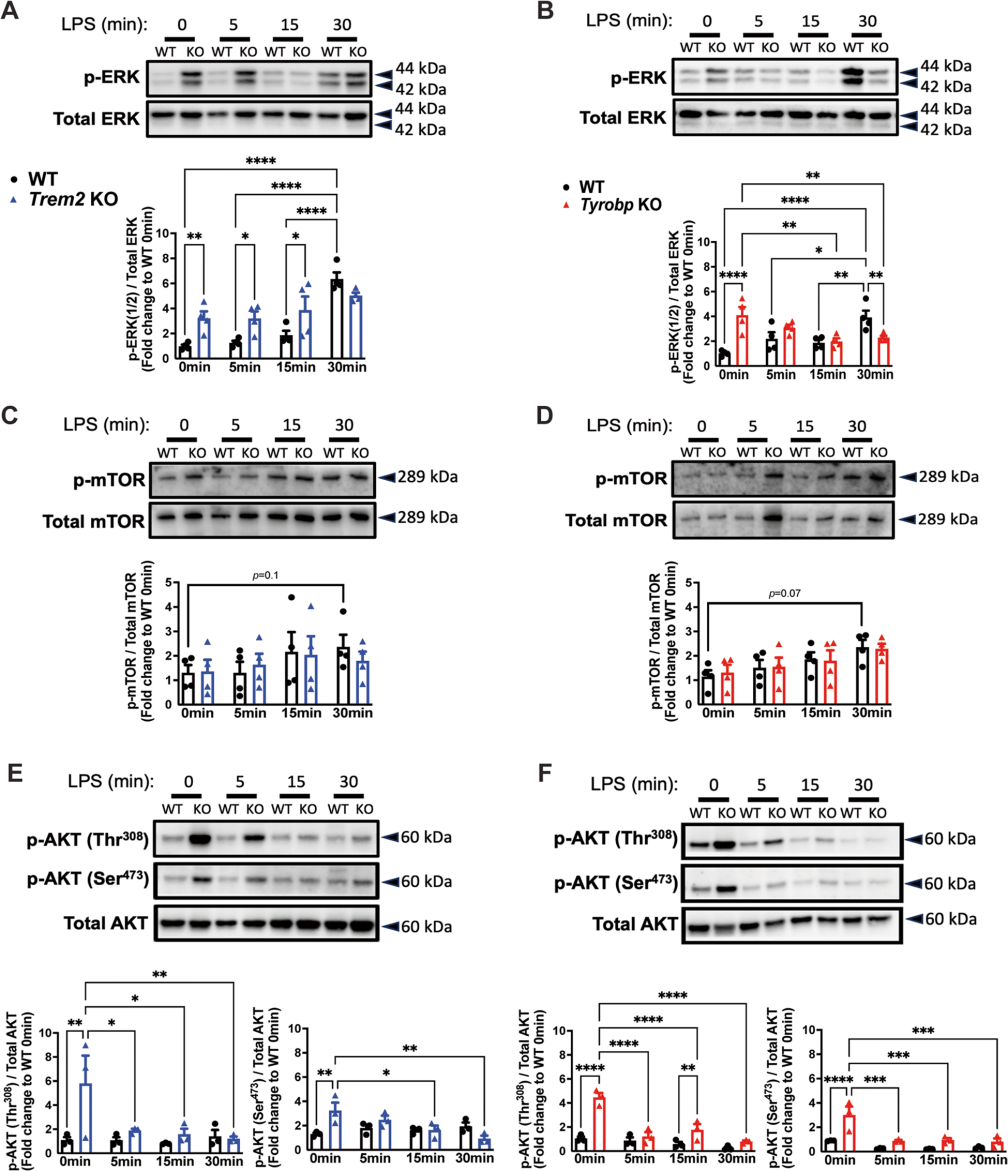
LPS stimulation fails to increase ERK phosphorylation in *Trem2* KO or *Tyrobp* KO microglia. **A)** Western blot and densitometry quantification of p-ERK and total ERK at baseline and after an LPS exposure of 5, 15 and 30 min for WT and *Trem2* KO microglia, and for **B)** WT and *Tyrobp* KO microglia. **C)** Western blot and densitometry quantification of p-mTOR and total mTOR at baseline and after an LPS exposure of 5, 15 and 30 min for WT and *Trem2* KO microglia, and for **D)** WT and *Tyrobp* KO microglia. **E)** Western blot and densitometry quantification of p-AKT (Thr^308^), p-AKT (Ser.^473^), and total AKT at baseline and after an LPS exposure of 5, 15 and 30 min for WT and *Trem2* KO microglia, and for **F)** WT and *Tyrobp* KO microglia. **A-F)** *N* = 4 independent experiments (data represented here is the average of all 4 experiments). Bars represent means ± SEM. Black bars and black-filled circles represent WT microglia, blue bars and blue-filled triangles represent *Trem2* KO microglia, and red bars and red-filled triangles represent *Tyrobp* KO microglia. Data were analyzed using a two-way ANOVA followed by Tukey’s multiple comparisons test. *p < 0.05. **p < 0.01. ***p < 0.001. ****p < 0.0001

To confirm the activation of primary microglia with LPS stimulation, we measured the expression of downstream signaling proteins in WT and KO microglia. WT microglia showed a time-dependent increase in ERK phosphorylation (p-ERK) when stimulated with LPS (Fig. 2A, B). Interestingly, compared to WT controls, baseline levels of p-ERK were increased in *Trem2* KO (Fig. 2A) and *Tyrobp* KO microglia (Fig. 2B). After LPS stimulation, however, p-ERK in *Trem2* KO microglia continued to remain elevated (Fig. 2A), while it significantly decreased in *Tyrobp* KO microglia, compared to baseline (Fig. 2B). Moreover, the mTOR signaling pathway was also investigated, and phosphorylation of mTOR (p-mTOR) trended toward increase after 30 min of LPS treatment in WT microglia (Fig. 2C, D). *Trem2* KO and *Tyrobp* KO microglia followed a similar trend as WT microglia (Fig. 2C, D).

Since mTOR is responsible for the phosphorylation of AKT, specifically at the Serine^473^ site, both AKT targets (Ser^473^ and Thr^308^) were assessed in primary microglia. Baseline phosphorylation status of AKT (Thr^308^) and AKT (Ser^473^) was significantly elevated in *Trem2* KO (Fig. 2E) and *Tyrobp* KO (Fig. 2F), when compared to WT microglia. After LPS stimulation, although there is no significant change in WT microglia over time, *Trem2* KO and *Tyrobp* KO microglia showed a decrease in AKT phosphorylation, compared to baseline (Fig. 2E, F).

Given that Syk phosphorylation is immediately downstream from the TREM2/TYROBP complex in microglia, we also measured phosphorylated protein levels of Syk at two different sites: namely, Tyrosine 525/526 in the activation loop of the Syk kinase domain and Tyrosine 352 in association with PLCγ (Supplementary Fig. 4 A, B). In WT primary microglia, as well as in *Trem2* KO and *Tyrobp* KO, there were no significant differences in p-Syk levels at baseline nor after a rapid LPS stimulation (Supplementary Fig. 4 A, B). Taken together, these results suggest a similar disrupted baseline activation in both *Trem2* KO and *Tyrobp* KO, compared to WT microglia, as summarized in Table 2. However, after LPS stimulation, only ERK phosphorylation showed different activation patterns, with decreasing p-ERK levels observed over time in *Tyrobp* KO microglia (opposite to WT microglia), while p-ERK levels in *Trem2* KO microglia did not respond to LPS stimulation.

**Table 2:** Summary of Fig. 2 and Supplementary Fig. 4

Protein Signaling Summary	WT	*Trem2* KO	*Tyrobp* KO
p-ERK at baseline	**⎯**	**↑**	**↑**
p-ERK after LPS	**↑**	No change from baseline	**↓**
p-mTOR at baseline	**⎯**	No change from WT control	No change from WT control
p-mTOR after LPS	**↑**	No change from baseline	No change from baseline
p-AKT at baseline	**⎯**	**↑**	**↑**
p-AKT after LPS	No change from baseline	**↓**	**↓**
p-Syk at baseline	**⎯**	No change from WT control	No change from WT control
p-Syk after LPS	No change from baseline	No change from baseline	No change from baseline

Baseline, up and down arrows indicate comparison of KO to WT at baseline. After stimulation, up and down arrows indicate comparison to baseline measurements for each respective genotype. Dash line indicates no comparison performed

### Aβ fibril stimulation increases ERK and mTOR phosphorylation in WT primary microglia, but not in *Trem2* KO or *Tyrobp* KO microglia

**Fig. 3:**
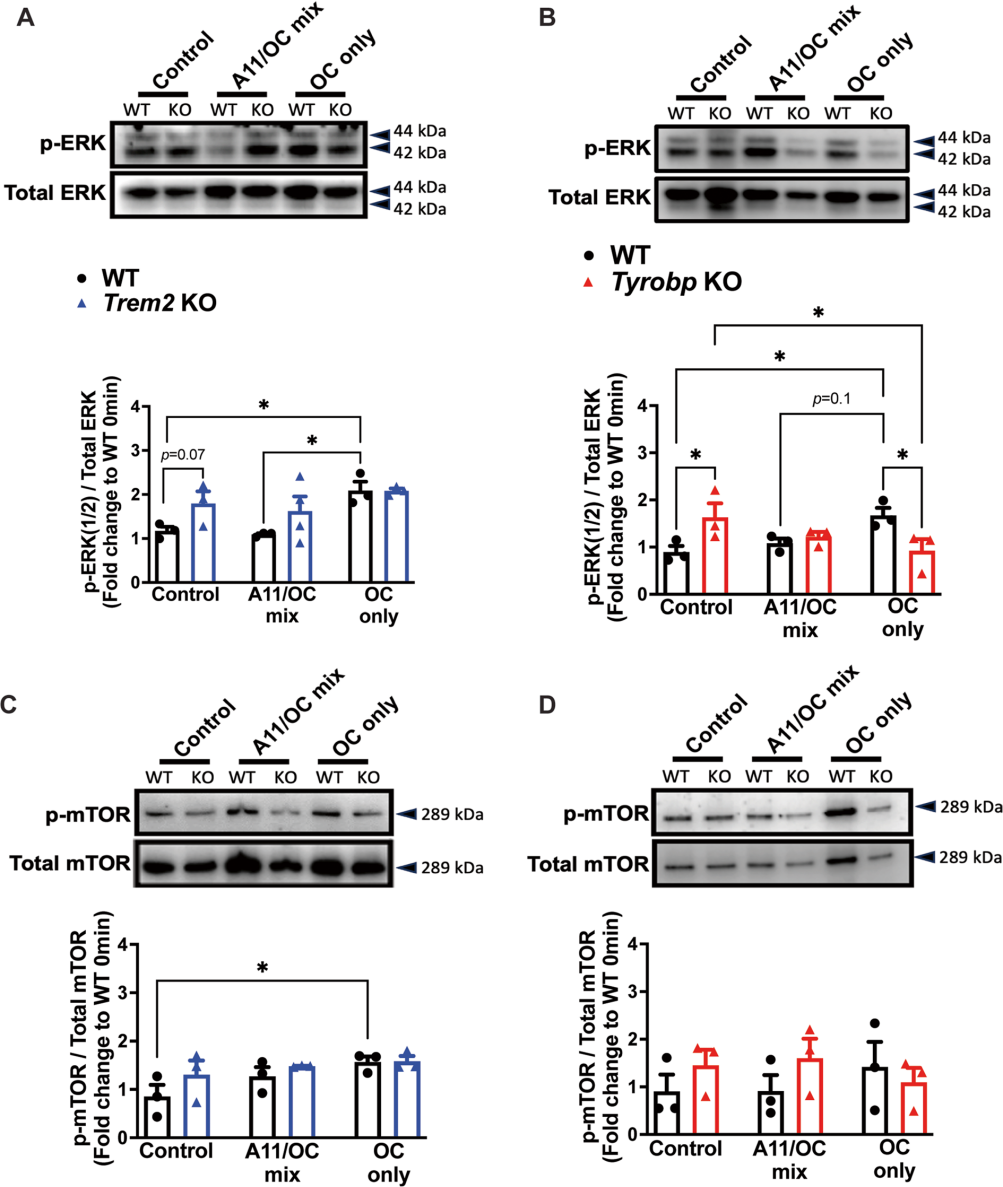
ERK activation differs in *Trem2* KO and *Tyrobp* KO microglia after Aβ fibril stimulation. **A)** Western blot and densitometry quantification of p-ERK and total ERK at baseline and after a 24-h stimulation with A11/OC mix or OC-only preparations from WT and *Trem2* KO microglia, and for **B)** WT and *Tyrobp* KO microglia. **C)** Western blot and densitometry quantification of p-mTOR and total mTOR at baseline and after a 24-h stimulation with A11/OC mix or OC-only preparations from WT and *Trem2* KO microglia, and for **D)** WT and *Tyrobp* KO microglia. **A-D)** *N* = 3 independent experiments (data represented here is the average of all 3 experiments). Bars represent means ± SEM. Black bars and black-filled circles represent WT microglia, blue bars and blue-filled triangles represent *Trem2* KO microglia, and red bars and red-filled triangles represent *Tyrobp* KO microglia. Data were analyzed using a two-way ANOVA followed by Tukey’s multiple comparisons test. *p < 0.05. **p < 0.01

To assess primary microglial activation in response to a mixture of Aβ oligomers and fibrils (A11 [prefibrillar oligomers]/OC [fibrils]) or fibrils alone (OC-only), protein expression and phosphorylation of downstream targets was measured in WT, *Trem2* KO and *Tyrobp* KO primary microglia after a 24-h stimulation (Fig. 3). Like LPS stimulation, ERK phosphorylation increased in WT primary microglia in response to a 24-h OC-only treatment (Fig. 3A, B). However, WT microglia showed no significant change in ERK phosphorylation in response to stimulation with a mixture of A11- and OC-type proteoforms (Fig. 3A, B). After a 24-h serum-free media incubation, in the absence of any stimulation, both *Trem2* KO and *Tyrobp* KO microglia still showed increased phosphorylation of ERK, compared to WT at baseline (Fig. 3A, B). *Trem2* KO microglia did not show a change in ERK activation, either following stimulation with either OC-only fibrils or with a mixture of A11- and OC-type Aβ prefibrillar oligomers, compared to KO controls (Fig. 3A). However, *Tyrobp* KO microglia exposed to OC-only Aβ fibrils showed a significant decrease in ERK phosphorylation, compared to KO controls (Fig. 3B).

Like ERK, mTOR phosphorylation increased in response to OC-only treatment in WT microglia compared to controls (Fig. 3C). Similar to what was observed after LPS stimulation, phosphorylation of mTOR did not change with OC-only treatment in either *Trem2* KO or *Tyrobp* KO microglia (Fig. 3C, D).

These results suggest that the baseline disruption of ERK phosphorylation seen in KO microglia after a rapid (30 min) incubation in serum-free unsupplemented media, persists in the absence of any stimulation, even after a 24-h incubation in serum-free unsupplemented media. Our results also suggest that differences between *Trem2* KO and *Tyrobp* KO microglia are more profound when exposed to fibrillar Aβ oligomers than when exposed to a mixture of pre-fibrillar/fibrillar Aβ oligomers (Table 3).

**Table 3:** Summary of Fig. 3

Protein Signaling Summary (24 h)	WT	*Trem2* KO	*Tyrobp* KO
p-ERK at baseline	**⎯**	**↑**	**↑**
p-ERK after Aβ fibrils	**↑**	No change from baseline	**↓**
p-mTOR at baseline	**⎯**	No change from WT	No change from WT
p-mTOR after Aβ fibrils	**↑**	No change from baseline	No change from baseline

Baseline, up and down arrows indicate comparison of KO to WT at baseline. After stimulation, up and down arrows indicate comparison to baseline measurements for each respective genotype. Dash line indicates no comparison performed

### Syk inhibition decreases ERK activation in *Tyrobp* KO at baseline, but not in *Trem2* KO microglia

**Fig. 4:**
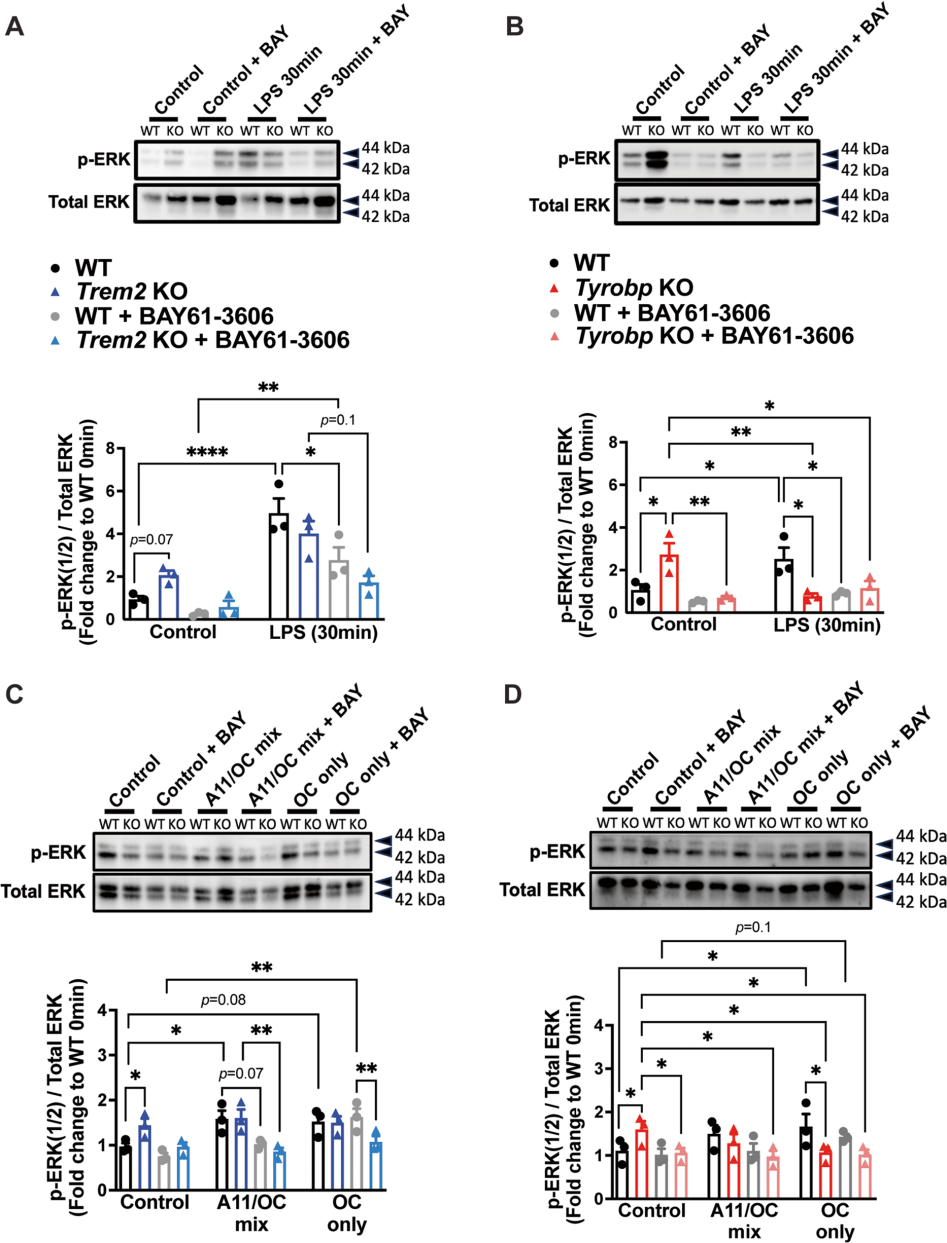
Syk inhibition decreased Erk phosphorylation at baseline in *Tyrobp* KO microglia, but not in *Trem2* KO microglia. **A)** Western blot and densitometry quantification for p-ERK and total ERK from WT and *Trem2* KO in the presence or absence of Syk inhibitor, BAY61-3606 (BAY), at baseline and after either a 30-min LPS stimulation, or after **B)** a 24-h stimulation with A11/OC mix or OC-only preparations. **C)** Western blot and densitometry quantification for p-ERK and total ERK from WT and *Tyrobp* KO in the presence or absence of BAY, at baseline and after either a 30-min LPS stimulation, or after **D)** a 24-h stimulation with A11/OC mix or OC-only preparations. **A-D)** *N* = 3 independent experiments (data represented here is the average of all 3 experiments). Bars represent means ± SEM. Black bars and black-filled circles represent WT microglia, blue bars and blue-filled triangles represent *Trem2* KO microglia, and red bars and red-filled triangles represent *Tyrobp* KO microglia. Data were analyzed using a two-way ANOVA followed by Tukey’s multiple comparisons test. *p < 0.05. **p < 0.01. ***p < 0.001. ****p < 0.0001

To analyze the involvement of Syk signaling in the downstream activation of protein targets in the absence of either TREM2 or TYROBP, primary microglia were pre-incubated for 4 h with BAY61-3606 (5µM), a highly selective inhibitor of Syk kinase [41], and then, stimulated with either LPS or Aβ prefibrillar oligomers or fibrils (Fig. 4). Pre-exposure to BAY61-3606 significantly decreased ERK phosphorylation after LPS (Fig. 4A, C), but not after an A11/OC mix and OC-only stimulation (Fig. 4B, D) in WT primary microglia. Syk inhibition also decreased ERK phosphorylation in *Trem2* KO after LPS and Aβ oligomer and fibril stimulation (Fig. 4A, B). Pre-incubation with BAY61-3606 led to a decrease of ERK phosphorylation in *Tyrobp* KO microglia at baseline to similar levels as when stimulated with LPS or Aβ stimulation (Fig. 4C, D). These results suggest that 24-h fibril stimulation in WT cells does not exclusively activate microglia through the Syk signaling pathway, or their activation overrides the possible Syk inhibition at the concentration used in this study. However, when cells lack TYROBP, Syk inhibition is sufficient to decrease Aβ activation, characterized in our studies by ERK phosphorylation. *Trem2* KO microglia exhibited the same directional trend without statistical significance. Therefore, our results suggest that *Tyrobp* KO rely on Syk activity to maintain ERK pathway activation at baseline.

### Rapid TLR4 inhibition decreases ERK activation in LPS-stimulated *Trem2* KO and *Tyrobp* KO primary microglia, while TLR4 inhibition increases ERK activation in WT microglia after 24 h

To evaluate whether the changes in ERK phosphorylation were primarily mediated by activation through the TREM2/TYROBP signaling axis, or whether there was an alternative pathway through the Toll-like receptor 4 (TLR4), primary microglia were pre-incubated for 4 h with a small molecule inhibitor of TLR4, TAK-242 (1μγ/mL) [42], and then stimulated with either LPS for 30 min or Aβ oligomers or fibrils for 24 h (Supplementary Fig. 1 and 5). At baseline, TLR4 inhibition decreased ERK phosphorylation in both *Trem2* KO and *Tyrobp* KO microglia (Supplementary Fig. 5A, B). After LPS stimulation, TLR4 inhibition decreased ERK phosphorylation in all of the groups (Supplementary Fig. 5A, B).

Intriguingly, contrary to what was seen in the shorter 4 h pre-treatment with TAK-242, WT primary cells show an increase in ERK phosphorylation after a 24 h treatment with TAK-242, in the absence of any other stimulation (Supplementary Fig. 5C, D). Similarly, long-term TAK-242 treatment led to an increase in ERK phosphorylation in *Trem2* KO microglia in the presence of Aβ stimulation (Supplementary Fig. 5C). However, the same treatment did not produce a significant change in ERK phosphorylation levels in *Tyrobp* KO microglia (Supplementary Fig. 5D). These results suggest that TAK-242 markedly reduced the microglial response to LPS, as expected, but it did not attenuate the response to the oligomers and fibrils mix or fibrils alone, indicating that the fibril-induced signaling is TLR4-independent and therefore unlikely to be driven by endotoxin contamination.

### PP1/PP2A inhibitor increases ERK activation in LPS-stimulated WT cells with no differences in *Trem2* KO or *Tyrobp* KO microglia

To determine whether protein phosphatases 1 and 2 A (PP1/PP2A) were involved in the baseline dysregulation in ERK phosphorylation that was observed in *Trem2* KO and *Tyrobp* KO primary microglia, cells were pre-treated with okadaic acid (OA), which is a marine toxin that inhibits PP1/PP2A [43] (Supplementary Fig. 1 and 6). In WT primary microglia, pre-incubation with OA increased ERK phosphorylation, after LPS treatment, to a similar extent (Supplementary Fig. 6A, B). In *Trem2* KO or *Tyrobp* KO microglia, however, pre-incubation with OA did not significantly alter p-ERK at baseline or after LPS stimulation, suggesting that PP1/PP2A inhibition does not further dysregulate baseline phosphorylation (Supplementary Fig. 6A, B). Overall, these results suggest that PP1/PP2A do not appear to be responsible for the baseline dysregulation of ERK phosphorylation that is observed in the absence of TREM2 or TYROBP. The inhibition results are summarized in Table 4.

**Table 4:** Summary of Fig. 4, Supplementary Fig. 5, and Supplementary Fig. 6

ERK Signaling Summary	WT	*Trem2* KO	*Tyrobp* KO
Baseline + BAY61-3606	No change from baseline	No change from baseline	**↓**
LPS + BAY61-3606	**↓**	**↓**	No change from LPS
Oligomers + BAY61-3606	No change from baseline	**↓** from A11/OC and OC-only stimulations	No change from baseline
Baseline + TAK-242	No change from baseline	**↓** from KO control	**↓** from KO control
LPS + TAK-242	**↓** from LPS stimulation	**↓** from LPS stimulation	No change from LPS stimulation
Oligomers + TAK-242	**↑** from baseline, A11/OC, and OC-only stimulation	**↑** from A11/OC, and OC-only stimulation	No change from A11/OC, or OC-only stimulation
Baseline + Okadaic Acid	**↑** from baseline	No change from baseline	No change from baseline
LPS + Okadaic Acid	No change from LPS stimulation	No change from LPS stimulation	No change from LPS stimulation

Baseline, up and down arrows indicate comparison of KO to WT at baseline, unless specified otherwise. After stimulation, up and down arrows indicate comparison to baseline measurements for each respective genotype, unless specified otherwise

### Lack of TREM2 or TYROBP showed large-scale changes in microglial gene expression at baseline

**Fig. 5:**
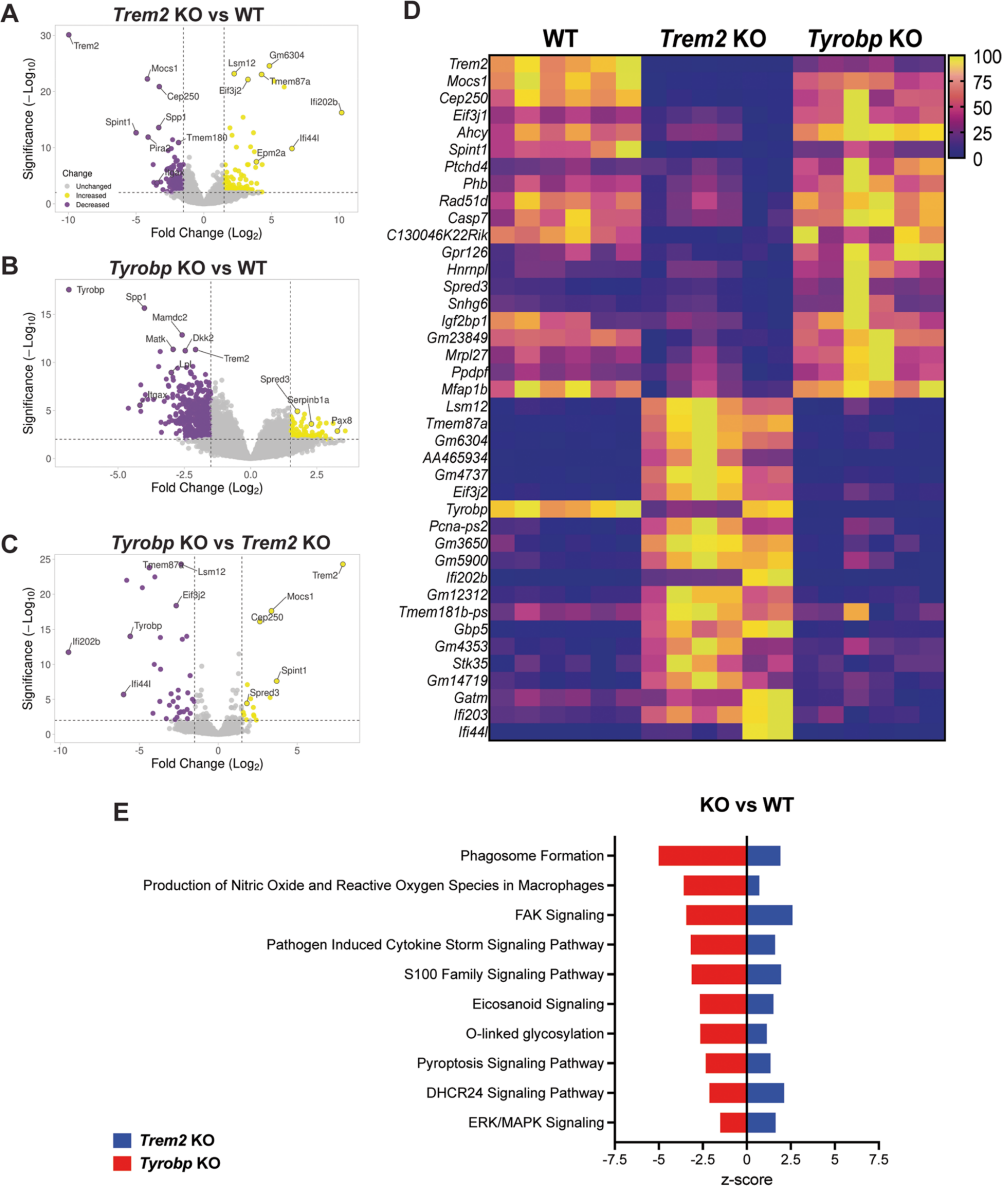
Lack of TREM2 or TYROBP showed large-scale changes in microglial gene expression at baseline. **A)** Volcano plot analysis identified upregulated (yellow) and downregulated (purple) genes at baseline (in the absence of any stimulation) in *Trem2* KO vs WT microglia, in **B)** *Tyrobp* KO vs WT microglia, and in **C)** *Tyrobp* KO vs *Trem2* KO microglia. Unchanged genes are depicted in gray. The annotated dots in the volcano plots are data points with a large (Manhattan) distance from the origin and are above the thresholds indicated by the dashed line. The threshold was set at 1.5-fold change and a significance level of 0.05. **D)** Heatmap is showing the top 20 upregulated (yellow) and top 20 downregulated (purple) DEGs for the *Tyrobp* KO vs *Trem2* KO comparison (WT counterparts are added for comparison). **E)** IPA results showing canonical pathways downregulated or upregulated in KO vs WT. *Trem2* KO is represented in blue, and *Tyrobp* KO is represented in red. Positive z-scores indicate pathways that are predicted to be activated (increased), while negative z-scores indicate pathways that are predicted to be inhibited (decreased). The higher the absolute z-score value, the stronger the prediction of activation or inhibition

Next, RNA sequencing was performed to determine the dysregulated genes and pathways in *Trem2* KO and *Tyrobp* KO primary microglia at baseline. There were 1190 downregulated DEGs and 1253 upregulated DEGs when comparing *Trem2* KO to WT microglia at baseline (Fig. 5A). Using a cutoff of FDR < 0.05, the top 20 upregulated and top 20 downregulated DEGs show striking differences at baseline between *Trem2* KO and WT microglia (Supplementary Fig. 7A). Well-established genes involved in microglial activation (*Spp1, Itgax, Rgs1, and Dok2*) were downregulated in *Trem2* KO compared to WT microglia (Supplementary Fig. 7A). *Epm2a*, which encodes laforin, a dual-specificity phosphatase that dephosphorylates glycogen [44], as well as genes involved in inflammatory signaling (*Gbp5, Ifi44l, Ifi202b*) were upregulated in *Trem2* KO microglia compared to WT (Supplementary Fig. 7A).

We additionally identified 2676 downregulated DEGs and 2288 upregulated DEGs when comparing *Tyrobp* KO to WT microglia at baseline (Fig. 5B). The top 20 upregulated and top 20 downregulated DEGs when comparing *Tyrobp* KO vs WT were quite different from the top 20 regulated DEGs detected in *Trem2* KO microglia (Supplementary Fig. 7B). Among the downregulated genes in *Tyrobp* KO microglia were those involved in migration and phagocytosis (*Mmp12, Atp6v0d2, and Ptger4*) (Supplementary Fig. 7B). Among the upregulated genes in *Tyrobp* KO microglia were genes that regulate cytokine signaling (*Htr2c*), immune interaction (*Cd209g*), and cell adhesion (*Mpzl2*) (Supplementary Fig. 7B).

Because most of the top upregulated and downregulated genes were dissimilar between *Trem2* KO and *Tyrobp* KO compared to WT, we also directly compared gene expression in *Trem2* KO and *Tyrobp* KO primary microglia at baseline, finding 99 downregulated and 77 upregulated DEGs (Fig. 5C). Genes that were upregulated in *Tyrobp* KO compared to *Trem2* KO were involved in microglial activation (*Casp7* and *Igf2bp1)*, MAPK signaling inhibition (*Spred3*), and metabolic regulation (*Ahcy, Mrpl27* and *Phb*), while the downregulated genes were involved in creatine metabolism and glycolysis (*Gatm*) and cytoskeletal organization (*Stk35*) (Fig. 5D).

We then selectively investigated homeostatic Stage 1 and Stage 2 disease-associated microglial (DAM1 and DAM2) gene expression in our dataset (Supplementary Fig. 7C-E). RNA levels from homeostatic genes, such as *Hexb*, *Cx3cr1*, *Ctsd*, *C1qa* and *C1qb*, were reduced in both *Trem2* KO and *Tyrobp* KO, compared to WT (Supplementary Fig. 7C). Interestingly, previous literature has shown that these homeostatic genes do not change their expression as a function of microglial transition in the context of AD [19], but we do see a change in *Trem2* KO and *Tyrobp* KO microglia, suggesting that TREM2 and TYROBP are essential for maintaining baseline microglial homeostasis, independent of disease-related activation. Expression of DAM1 genes, such as *Apoe* and *B2M*, was downregulated in both *Trem2* KO and *Tyrobp* KO microglia, compared to WT at baseline (Supplementary Fig. 7D). Since these microglia were not stimulated, this would suggest either that both TREM2 and TYROBP are required for normal expression of *Apoe* and *B2M* and/or that ablation of either *Trem2* or *Tyrobp* is sufficient to prevent microglial transition into DAM1, in the absence of disease-related stimuli. However, *Cstb*, *Fth1* and *Lyz2* were only downregulated in *Tyrobp* KO compared to WT, but not in *Trem2* KO microglia (Supplementary Fig. 7D). This would suggest that, at baseline, these genes are involved in a Trem2-independent but Tyrobp-dependent transition into DAM1. Since TYROBP is the key adaptor protein for multiple immune receptors (not just TREM2), this could also indicate that these genes require broader immune signaling mechanisms beyond TREM2 alone. DAM2 genes, such as *Ctsl*, *Lpl*, *Cd9*, *Ccl6*, *Itgax*, *Clec7a*, and *Lilrb4*, were downregulated in both *Trem2* KO and *Tyrobp* KO (Supplementary Fig. 7E), suggesting that both TREM2 and TYROBP are required for full microglial transition into the DAM2 stage. *Cst7* was downregulated and *Csf1* was upregulated only in *Tyrobp* KO (Supplementary Fig. 7E). Previous studies indicate that both *Csf1* and *Cst7* are upregulated in DAM2 [19, 20, 45], suggesting that *Cst7* might be activated in a TYROBP-dependent manner.

Ingenuity Pathway Analysis was performed to identify similar or different canonical pathways in *Trem2* KO or *Tyrobp* KO microglia (*p*-value < 0.05). The top canonical pathways enriched at baseline that show differences between *Trem2* KO and *Tyrobp* KO, compared to WT, are depicted in Fig. 5E. Interestingly**,** among the canonical pathways that showed the greatest differences, “Phagosome Formation” and “FAK signaling” had the highest inhibition z-score in *Tyrobp* KO, while they were among the highest activation z-score in *Trem2* KO microglia (Fig. 5E), suggesting differences in cell phagocytosis, proliferation and migration between genotypes. Similarly, “Pyroptosis signaling pathway” and “ERK/MAPK signaling” have substantial differences between *Trem2* KO and *Tyrobp* KO, suggesting higher inhibition of inflammatory cell death and ERK signaling in *Tyrobp* KO, and higher activation of those pathways in *Trem2* KO cells at baseline (Fig. 5E). Likewise, “DHCR24 Signaling Pathway” and “Eicosanoid Signaling” pathway enrichment suggests inhibition of lipid metabolism and immune signaling in *Tyrobp* KO, while these pathways were activated in *Trem2* KO microglia, when compared to WT (Fig. 5E). A summary of IPA-identified pathways and genes at baseline is depicted in Supplementary Table 2.

Comparison of our *Trem2* KO transcriptomic signatures at baseline with those from acutely isolated adult *Trem2* KO (GSE130627) [46] CD11b + microglia, revealed limited overlap by Venn Diagram analysis (Supplementary Fig. 8A). At the single-gene level, there were only 14 DEGs across both studies (Supplementary Fig. 8B). Rank-rank scatter plot analysis showed modest global agreement in effect directions, but not strong gene-by-gene corcondance (Supplementary Fig. 8C). However, this comparison showed consistent alignment at the pathway level, particularly within inflammatory and lipid-sensing signaling (Reactome Pathways 2024, Supplementary Fig. 8D).

### Pronounced and comparable differential gene expression signatures between *Trem2* KO and *Tyrobp* KO after stimulation with LPS or Aβ fibrils, but not after stimulation with an Aβ oligomer/fibril mixture

**Fig. 6:**
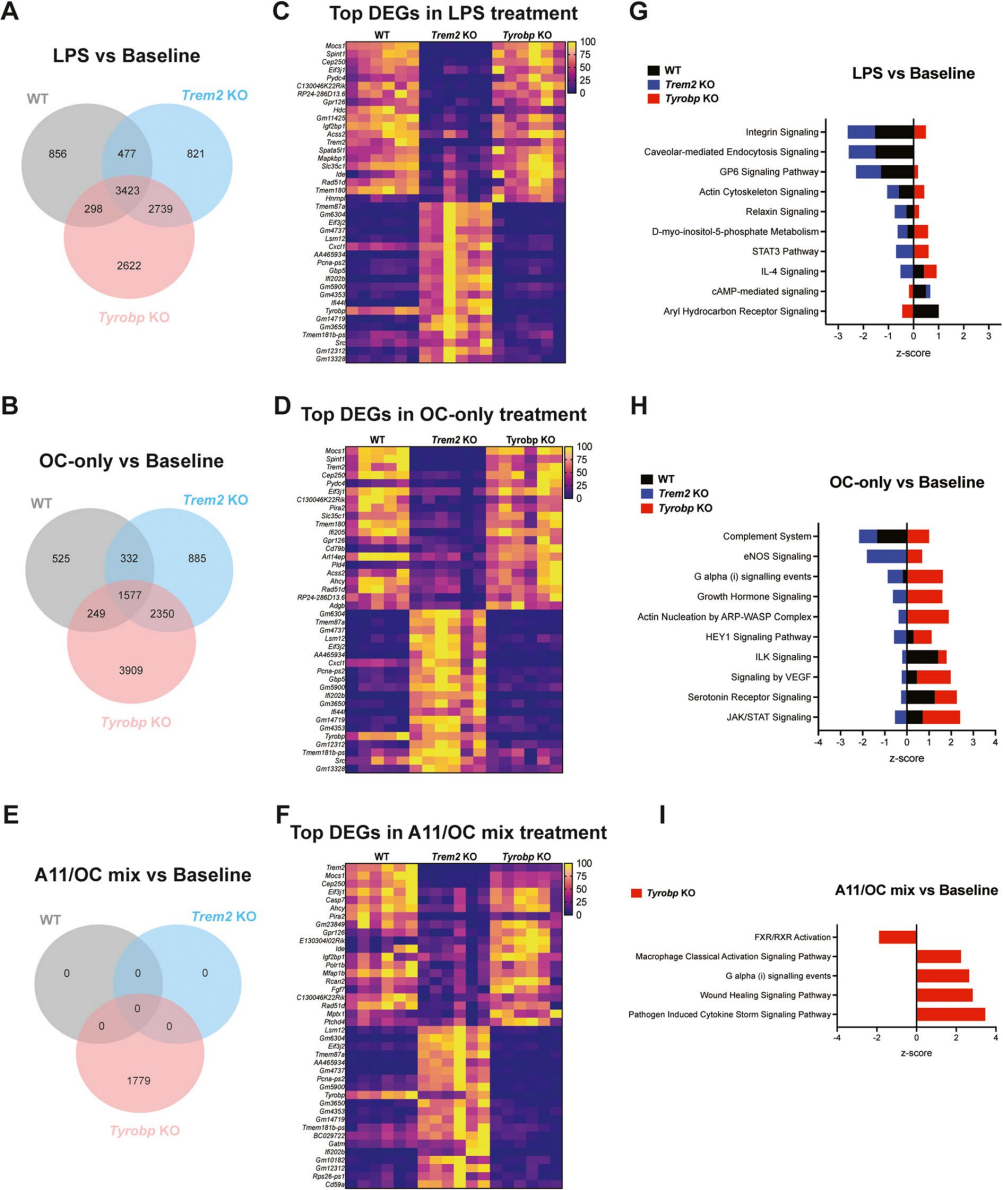
Lipid signaling and inflammatory signature distinctions between *Trem2* KO and *Tyrobp* KO microglia. **A)** Venn diagram of DEGs between LPS stimulation and baseline conditions for WT, *Trem2* KO, and *Tyrobp* KO microglia. **B)** Venn diagram of DEGs between OC-only stimulation and baseline conditions for WT, *Trem2* KO, and *Tyrobp* KO microglia. **C)** Heatmaps showing the top 20 upregulated and top 20 downregulated DEGs that are changed in *Tyrobp* KO microglia compared to *Trem2* KO microglia in response to a 24-h LPS stimulation or **D)** OC-only stimulation. **E)** Venn diagram of DEGs between A11/OC mix stimulation and baseline conditions for WT, *Trem2* KO, and *Tyrobp* KO microglia. **F)** Heatmap showing the top 20 upregulated and top 20 downregulated DEGs that are changed in *Tyrobp* KO microglia compared to *Trem2* KO microglia in response to a 24-h A11/OC mix stimulation. **G)** IPA results showing predicted activation or inhibition z-scores of canonical pathways when comparing LPS vs. baseline, H) OC-only vs. baseline, or I) A11/OC vs. baseline. WT is represented in dark gray/black, Trem2 KO is represented in blue, and *Tyrobp* KO is represented in red

We also performed RNA sequencing in WT, *Trem2* KO, and *Tyrobp* KO primary microglia after cells were stimulated with either LPS, Aβ oligomer/fibril mix, or Aβ fibril-only for 24 h. To find differences and similarities, *Tyrobp* KO vs *Trem2* KO DEGs were compared among the different stimulations (Fig. 6). Venn analysis showed that a similar number of genes was differentially expressed after LPS and OC-only stimulation (Fig. 6A, B). Many of the top 20 upregulated and downregulated DEGs were driven by the genotype differences, rather than the specific treatments (Fig. 6C, D, F). However, among genes upregulated in *Tyrobp* KO and downregulated in *Trem2* KO after LPS stimulation were *Hdc* (involved in inflammation and ramification of microglia), *Spata5l1* (linked to mitochondrial function, and affects cell growth, migration, and proliferation), and *Mapkbp1* (regulates the JNK signaling) (Fig. 6C). Among the genes upregulated in *Tyrobp* KO and downregulated in *Trem2* KO microglia after Aβ fibril-only stimulation were *Ifi205* (acts within cellular response to IFN-β) and *Pld4* (phospholipase D4 colocalizes with phagosomes) (Fig. 6D).

Interestingly, the mix of Aβ oligomer/fibril did not stimulate primary microglia to the same extent as LPS or Aβ fibrils alone (Fig. 6E). When using Venn diagrams to show the overlap among the DEGs derived from WT, *Trem2* KO, and *Tyrobp* KO after A11/OC treatment compared to baseline, we found no significant DEGs in WT and *Trem2* KO, but 1779 DEGs were found in *Tyrobp* KO (Fig. 6E). When comparing DEGs between *Trem2* KO and *Tyrobp* KO in the A11/OC treatment, *Tyrobp* KO microglia showed upregulation of *Fgf7* (involved in microglia activation without cell death), and *Rcan2* (negative regulator of calcineurin), while showing downregulation of genes altered in neurodegeneration (e.g. *CD59a*) (Fig. 6F). Our results suggest that TYROBP deficiency alters microglial lipid metabolism and phagocytosis in response to fibrillar Aβ, uniquely modifying inflammatory and metabolic pathways, leading to distinct microglial activation states, compared to *Trem2* KO microglia.

### Differences in lipid signaling and inflammatory signatures between *Trem2* KO and *Tyrobp* KO microglia

To understand the potential functional impact of the identified DEGs upon stimulation, IPA canonical pathway analyses were performed in our study (Fig. 6G-I). After LPS stimulation, canonical pathways such as “Integrin signaling”, “GP6 signaling pathway”, “Actin cytoskeleton signaling,” and “D-myo-inositol-5-phosphate metabolism” show a predicted inhibition in *Trem2* KO microglia, and a predicted activation z-score in *Tyrobp* KO microglia (Fig. 6G). Interestingly, after LPS stimulation, canonical pathway “STAT3 pathway” was inhibited in *Trem2* KO and activated in *Tyrobp* KO, while it remained unchanged in WT microglia (Fig. 6G). This suggests an intrinsic difference in membrane dynamics after LPS stimulation in microglia that lack either TREM2 or TYROBP compared to WT.

After Aβ fibril-only treatment, “Complement system” pathway was *inhibited* in WT and *Trem2* KO microglia, while it was *activated* in *Tyrobp* KO (Fig. 6H). Canonical pathways “eNOS signaling”, “Growth hormone signaling” and “Actin nucleation by ARP-WASP complex” were unchanged in WT microglia after Aβ fibrils, but they were inhibited in *Trem2* KO and activated in *Tyrobp* KO microglia (Fig. 6H). This highlights the differential microglial activation modulation, cytoskeletal remodeling, migration, and inflammatory response to Aβ fibrils when microglia lack either TREM2 or TYROBP.

Additionally, when *Trem2* KO microglia were stimulated with the A11/OC mix, no canonical pathways or diseases and functions pathways were identified based on the calculated z-scores and -log(p-values). However, *Tyrobp* KO canonical pathways analysis showed that the most activating pathways involved “Pathogen induced cytokine storm signaling pathway” and “Wound healing signaling pathway”, while the most inhibiting pathway involved “FXR/RXR activation” (Fig. 6I). This suggests that WT and *Trem2* KO microglia fail to respond to an Aβ oligomer/fibril mix, but *Tyrobp* KO microglia, despite some transcriptomic changes, still retain some capacity for functional responses to the Aβ oligomer/fibril mix.

Our results, in response to ‘neuroinflammation-like’ or ‘AD-like’ stimuli, suggest that *Trem2* KO microglia have suppressed cholesterol metabolism and immune responses, while *Tyrobp* KO microglia have overactive complement and lipid metabolism, potentially leading to dysregulated inflammatory responses. A summary of IPA-identified pathways and genes after stimulations is depicted in Supplementary Table 3.

### *Trem2* KO and *Tyrobp* KO microglia show differential proteomic signatures for pro-inflammatory activation and ECM-associated proteins

**Fig. 7:**
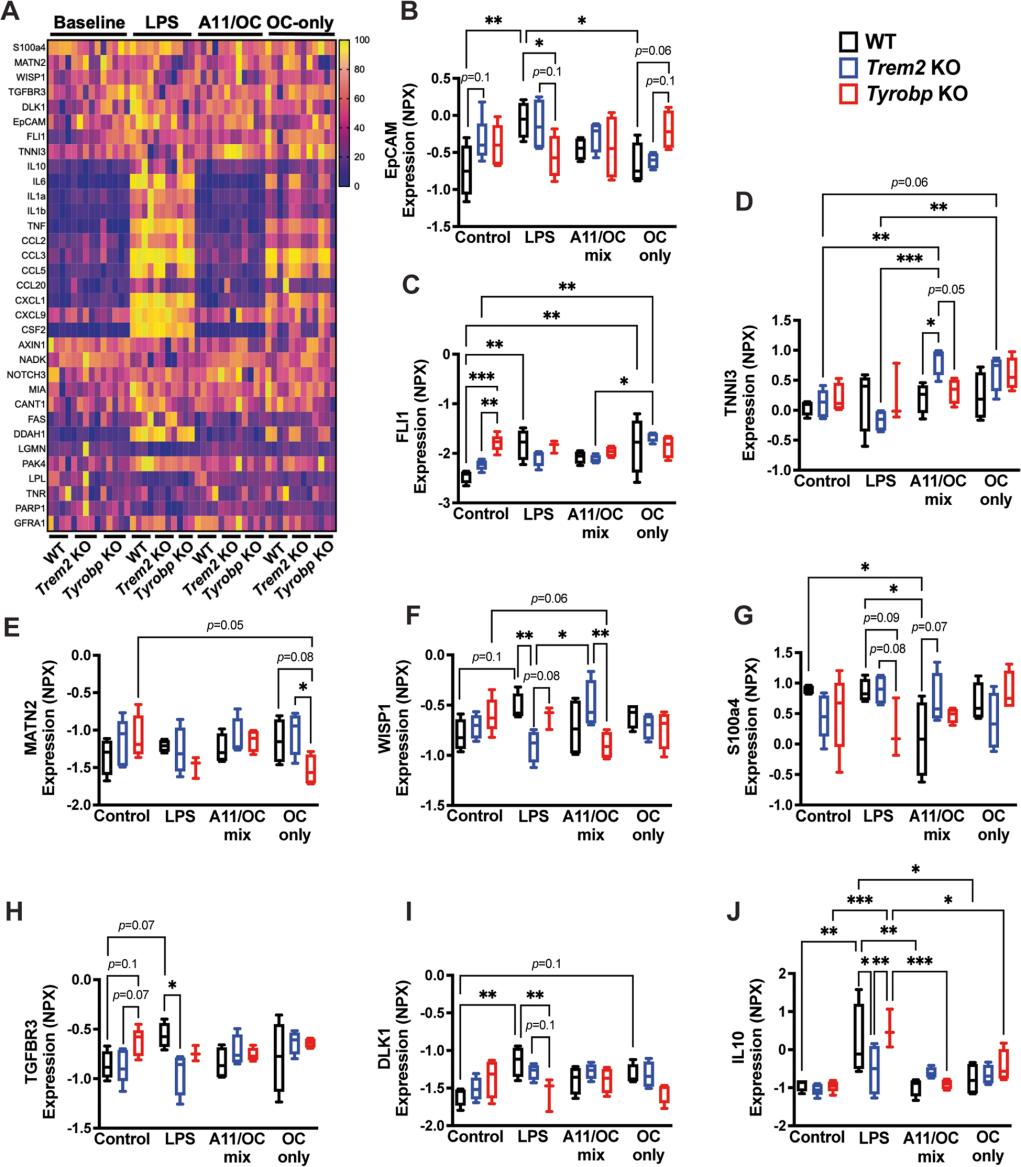
*Trem2* KO and *Tyrobp* KO show differential proteomic signatures for pro-inflammatory activation and ECM-sensing and remodeling. **A)** Heatmap of differentially expressed proteins that were increased (yellow) or decreased (purple) by either LPS, A11/OC mix, or OC-only stimulation, in conditioned media from WT, *Trem2* KO, and *Tyrobp* KO primary microglia. Values were normalized to show the smallest value as 0 and the largest value as 100. **B)** Dot plot showing NPX-values of EpCAM, **C)** FLI1, **D)** TNNI3, **E)** MATN2, **F)** WISP1, **G)** S100a4, **H)** TGFBR3, **I)** DLK1, and **J)** IL-10. **A-J)** *n* = 3–5 per genotype per condition. Black bars represent WT microglia, blue bars represent *Trem2* KO microglia, and red bars represent *Tyrobp* KO microglia. Data were analyzed using a two-way ANOVA followed by Tukey’s multiple comparisons test. *p < 0.05. **p < 0.01. ***p < 0.001. ****p < 0.0001

To broaden the proteomic signature of microglia, analysis using an Olink proteomics panel was performed on conditioned media from primary microglia treated with either LPS, A11/OC mix or OC-only for 24 h. Out of the 92 proteins included in the panel, a total of 9 proteins showed a significant Genotype x Treatment interaction, 5 proteins showed a significant main effect of genotype, and 20 proteins showed a significant main effect of treatment based on two-way ANOVA (Fig. 7). A summary of these molecules (total of 92) is depicted in Supplementary Table 4.

The expression heatmap shows the significant changes among the different groups (Fig. 7A). At baseline, EpCAM levels were increased in *Trem2* KO (Fig. 7B), while Fli1 was increased in *Tyrobp* KO (Fig. 7C), compared to WT controls. These proteins have been reported to be involved in the regulation of cell migration and proliferation, suggesting that baseline differences seen between *Trem2* KO and *Tyrobp* KO microglia (Fig. 1E) could potentially be linked to a dysregulation in the protein expression of these two molecules. Additionally, TNNI3, which has been reported as an upstream regulator of ion flux (Fig. 7D), levels were significantly increased in *Trem2* KO after an Aβ oligomer/fibrils mix. Because TNNI3 is an intracellular protein, its increased level in conditioned media suggests stimulus-dependent differences in microglial integrity or vesicle release in *Trem2* KO microglia.

Classic extracellular matrix (ECM)-associated proteins, such as WISP1 and MATN2, showed a decrease in response to Aβ oligomer/fibrils mix in *Tyrobp* KO, when compared to *Trem2* KO microglia (Fig. 7E, F). Another ECM-associated protein, S100a4, showed a decrease in response to LPS stimulation in *Tyrobp* KO conditioned media (Fig. 7G). Because these ECM-associated proteins modulate key cellular functions, such as adhesion, migration, proliferation, and matrix remodeling, their altered secretion patterns may underlie the distinct functional responses observed between *Trem2* KO and *Tyrobp* KO when challenged with different stimuli.

Interestingly, under basal conditions, TGFBR3, which has been shown to exacerbate AD pathology in mice and increased levels have been associated with AD in humans [47, 48], trended to be increased in *Tyrobp* KO, compared to WT and *Trem2* KO (Fig. 7H). TGFBR3 increased in response to LPS in WT microglia media, but its levels did not change in response to any stimulation in neither *Trem2* KO microglia nor *Tyrobp* KO microglia (Fig. 7H). The activated state in *Tyrobp* KO microglia after LPS stimulation might be linked to a decrease in DLK1 (Fig. 7I), which is a non-canonical inhibitor of NOTCH1 signaling.

Other studies have reported that IL-10 deficiency leads to a pro-inflammatory microglial phenotype [49]. In our study, IL-10 protein expression in *Trem2* KO microglia conditioned media was lower than both WT and *Tyrobp* KO microglia after LPS treatment (Fig. 7J), indicating that *Trem2* KO microglia are in a more pro-inflammatory state compared to WT and *Tyrobp* KO microglia. Moreover, IL-10 levels in WT and *Tyrobp* KO conditioned media only increased in response to LPS, but not in response to Aβ stimulation, suggesting that this response is TLR4-dependent.

Expression levels of CCL2, CCL3, CCL5, TNF, IL1a, IL1b, CSF2, IL6, CXCL1, and DDHA1 were increased in response to LPS and OC-only stimulation, but there were no genotype differences between the groups (Supplementary Fig. 9). The proteomics results from primary microglia conditioned media suggested basal differences in protein expression related to cell migration and cell proliferation between *Trem2* KO and *Tyrobp* KO. Moreover, 24-h LPS and Aβ-proteoforms stimulations reduced expression of pro-inflammatory proteins in *Trem2* KO and ECM-associated proteins in *Tyrobp* KO.

## Discussion

Our study has uncovered previously unrecognized mechanisms involved in microglial homeostasis and activation in response to inflammatory and AD-related stimulation. We determined the functional and biochemical outcomes of primary microglia that did not express either *Trem2* or *Tyrobp*, following treatment with either LPS, Aβ fibrils, and/or an Aβ oligomer/fibril mixture. This study demonstrated that there were intrinsic differences in transcriptomic and proteomic signatures between *Trem2* KO and *Tyrobp* KO microglia at baseline, and after stimulation, suggesting that TREM2 and TYROBP, which associate on the cell surface to trigger downstream signal transduction, each regulate essential microglial functions in distinctive ways.

It has been well-established that the binding of TREM2 to TYROBP initiates downstream signaling via PI3K and ERK, which thereby regulate microglial function [50], but the mechanisms by which this occurs remain largely unknown. Our study has shown that, at baseline, both *Trem2* KO and *Tyrobp* KO microglia have dysregulated levels of p-ERK, but the underlying transcriptional signatures that are related to the ERK/MAPK canonical signaling pathways are different. For instance, in *Trem2* KO microglia, the predicted activation of the ERK/MAPK signaling pathway was due to an increase in phospholipase A2 (*Pla2g2f, Pla2g4d, Pla2g4f*) gene expression, while the predicted inhibition of the ERK/MAPK signaling pathway in *Tyrobp* KO microglia was related to reduced phospholipase C gamma 2 (*Plcg2*) mRNA levels. Interestingly, both phospholipases are critical for microglial function, but they operate through distinct mechanisms and signaling pathways. Phospholipase A2 (PLA_2_) is important in the production of inflammatory mediators and is involved in the regulation of membrane-cytoskeleton connectivity and actin rearrangement in microglia [51–53]. PLA_2_ is also activated by phosphorylation through MAPK [53] and is upregulated in maladaptive activated microglia, but not in homeostatic microglia [52, 54]. However, $PLC\gamma 2$ is crucial for microglial activation and signaling, affecting functions like microglial viability, phagocytosis, and cholesterol metabolism [55, 56]. Genetic deficiency of $PLC\gamma 2$ in human macrophages and microglia results in reduced phagocytosis and reduced survival [56, 57], indicating that the decreased *Plcg2* mRNA levels that we detected in *Tyrobp* KO microglia might lead to the abnormal cellular responses at baseline. Furthermore, in relation to the PI3K/AKT canonical signaling pathway, both *Trem2* KO and *Tyrobp* KO microglia had a downregulation of various integrin subunits (*itgal*, *itgam*, *itgax*, *itgb2*) at baseline, compared to WT microglia. The expression of these integrins (CD11a, CD11b, CD11c, and CD18) is regulated by PARP-1, which has been shown to negatively regulate AKT [58, 59]. Therefore, the observed integrin downregulation in KO microglia may be mechanistically linked to the enhanced AKT phosphorylation detected at baseline.

The loss or alteration of TREM2 in microglia, moreover, impairs phagocytosis of Aβ [60], but details of how this process is regulated remain incompletely understood. In this study, we found decreased phagocytosis of green fluorescent beads at baseline in both *Trem2* KO and *Tyrobp* KO microglia compared to WT, but the transcriptional signatures related to the baseline dysregulation in phagosome formation were different. Specifically, in *Trem2* KO microglia, phagosome formation was related to increased expression of genes involved in actin binding (*Myh4*, *Myh7b*). In general, the head of myosin interacts with actin providing the required force for cell phagocytosis and movement [61]. Although our understanding of MYH4 and MYH7b function in microglia is incomplete, it has been reported that different type II myosins have diverse cellular responses. Melo et al*.* showed that both MYH9 and MYH10 affect microglial phagocytosis but play different roles: MYH9 regulates cell shape while MYH10 controls cell activation [62]. Similarly, MYH4 and MYH7b could potentially decrease phagosome formation in *Trem2* KO microglia by engaging different mechanisms that regulate myosin motor activity, which would require further exploration. In *Tyrobp* KO microglia, however, the decrease in phagosome formation was associated with a decrease in the levels of mRNAs encoding complement molecules (*C3*, *C3ar1, C5ar1, C5ar2*), toll-like receptors (*Tlr1, Tlr4, Tlr7, Tlr8*) and chemokine receptors (*Ccr2*, *Ccrl2*). In AD mouse models, early microglial activation, during the formation of Aβ plaques, appears to be beneficial, while after prolonged activation, microglial phagocytosis and clearance of plaques is reduced [63, 64]. Of note, acute C3/C3aR1 disruption decreases phagocytosis, while prolonged (1–2 days) C3/C3aR1 inhibition protects microglial phagocytic function [31, 65]. Similarly, other groups have suggested that pharmacologic or genetic disruption of C5aR1 in mouse AD models initially results in a reduction in phagocytic microglia, but if Aβ deposits have already formed, disease progression continues [63, 66]. Likewise, pharmacologic and genetic disruption of TLR4 inhibits microglial phagocytosis of axon debris in vivo [67], and *Ccr2*-deficiency impairs phagocytosis in vitro [68]. Taken together, the decrease we detected in TLR, complement, and chemokine gene expression in *Tyrobp* KO microglia could drive their decreased phagocytic capacity.

After LPS stimulation, we further found that predicted dysregulation in membrane lipid signaling differed between *Trem2* KO and *Tyrobp* KO microglia. LPS is an endotoxin and major component of the cell wall of Gram-negative bacteria that induces a “neuroinflammatory” state by increasing the release of pro-inflammatory cytokines, such as TNFα, IL-6 and IL-1β [69]. Previous studies in the microglial cell line BV2 have reported increased *Tlr4* expression as well as decreased *Trem2* expression after a 24-h LPS treatment [70]. TREM2 itself has been shown to negatively regulate TLR-induced responses [71]. In our study, *Trem2* KO microglia exposed to LPS for 24 h showed a predicted inhibition of membrane lipid signaling which was related to an increase in inositol polyphosphate-5-phosphatase B (*Inpp5b*) levels, and decreased expression of other phosphatase genes that regulate cell proliferation, growth, and differentiation (*Pawr*, *Ptpn13, Dusp13b, Ppp2r2c)*. INPP5B is involved in phagocytic cup formation and closure in hippocampus of LPS-treated mice [72], while PAWR plays a role in calcium signaling pathways [73], PTPN13 modulates tau tyrosine phosphorylation [74], DUSP13B functions in innate immunity during LPS-induced signaling [75], and PPP2R2C regulates PP2A activity and tau dephosphorylation, and is downregulated in aged AD mouse brains [76]. In *Tyrobp* KO microglia, the predicted activation of membrane lipid signaling was related to an increased expression of several phosphatase genes (*Ptpn1*, *Ptpn12*, *Ssh3, Sgpp2*), and decreased levels of mRNAs encoding phosphatases that are involved in the regulation of heparan sulfate proteoglycan biosynthesis (*Pxylp1*) and those involved in the cell cycle (*Cdc25b, Cdc25c*, *Ppp2r2b*). For instance, PTP1B is a positive regulator of neuroinflammation through activation of Src kinase by dephosphorylation at the membrane [77], which in our study could potentially initiate the signaling dysregulation in *Tyrobp* KO microglia. Moreover, the slingshot protein phosphatase 3 (SSH3) regulates actin dynamics by promoting actin turnover [78], suggesting that increased *ssh3* expression in *Tyrobp* KO microglia might have dysregulated cell shape and motility. In contrast, downregulation of *Pxylp1*, which modifies proteoglycans, could potentially influence cell adhesion, migration and signaling in *Tyrobp* KO microglia after LPS stimulation. Recent human genetic evidence has identified a monoallelic TYROBP deletion as a risk factor for late-onset AD and it was associated with Nasu Hakola Disease (NHD)-type bone cysts in a subset of carriers [79]. Mechanistically, the study reports reduced TYROBP protein levels in myeloid cells, and an exaggerated inflammatory transcriptional response to LPS in monocyte-derived microglia-like cells. These human data are consistent with our findings that perturbing the TREM2/TYROBP axis reconfigures the inflammatory signature in microglia, and underscore that TYROBP dosage (not only complete loss) can shift microglial reactivity.

Microglia have also been reported to recognize oligomeric and fibrillar Aβ and respond by increasing the release of proinflammatory cytokines and reactive oxygen species [80]. In our study, comparison of *Trem2* KO and *Tyrobp* KO responses to fibrils-only stimulation revealed different regulatory signatures of microglial inflammatory signaling (complement system, JAK/STAT, G alpha (i), ILK, VEGF, and eNOS signaling). In *Trem2* KO microglia, predicted inhibition of microglial inflammatory signaling was related to a decrease in the expression of genes involved in lipid metabolism, including actin cytoskeleton reorganization genes (*Rap2a*, *Kras*), and cell membrane integrity genes (*Prkd1*), and a decrease in complement gene expression (*C1qa, C1qb, C1qc)*. Although studies in microglia are limited, Rap2a in macrophages is activated by LPS, and its modulation disrupted the production of important inflammatory mediators [81], suggesting an important role in the inflammatory dysregulation in *Trem2* KO microglia that we observed in response to Aβ fibrils. Similarly, PKD-mediated signaling pathways have been coupled with morphology, migration and inflammatory responses in BV2 cells and primary microglia in other studies [82], suggesting that the downregulation of *Prkd1* in *Trem2* KO might influence these microglial functions. Interestingly, in human AD subjects, complement gene expression (*C1qa, C1qb, C1qc)* is upregulated in microglia, but only in a subpopulation that was smaller in proportion in AD patients [83]. The decrease in this microglial subpopulation was suggested to contribute to the imbalance of complement signaling seen in AD [83]. Those results are in line with the decrease in complement gene expression we observed in Aβ fibril-stimulated *Trem2* KO microglia in this study. In contrast, in *Tyrobp* KO microglia, activation of the microglial inflammatory signaling signature was related to increased levels of mRNAs encoding protein kinases (*Prkcb*, *Prkch, Pik3cd),* and complement molecules (*C2*, *C3ar1)*. PRKCB protein levels have been reported to be low in human patients with AD, potentially disrupting phagocytosis, gap junctions and MAPK signaling pathways [84]. In our study, however, exposure of *Tyrobp* KO microglia to Aβ fibrils for 24 h resulted in increased *Prkcb* expression, suggesting that the upregulation may occur as a compensatory mechanism, or that it reflects an alternative activation state distinct from the chronic dysregulation seen in AD. The upregulation of complement-related genes in *Tyrobp* KO microglia upon Aβ fibril exposure possibly occurs to either compensate for impaired phagocytosis or induce a pro-inflammatory response and suggests that TYROBP normally suppresses excessive complement activation.

Validating differences in migration we observed between *Trem2* KO and *Tyrobp* KO microglia under basal conditions, our study revealed two molecules (EpCAM and FLI1) that are involved in cell migration and proliferation. Although the functional role(s) that EpCAM plays in microglia remain to be fully elucidated, EpCAM is expressed in microglia and has been implicated in cell adhesion, migration, and inflammatory responses [85, 86]. Our results suggest that the increase in EpCAM protein levels at baseline in the absence of functional TREM2 may be an attempt to maintain cellular integrity and prioritize cell adhesion and survival over migration, in the presence of increased inflammation or dysfunction. Similarly, the role of FLI1 in microglial migration is also understudied, however, FLI1 is known to enhance PI3K/AKT signaling via the negative regulation of the inositol phosphatase SHIP-1 in other cell types [87]. PI3K/AKT signaling is also known to modulate microglial migration [88]. Consistent with these studies, the modest increase in cell migration we observed in *Tyrobp* KO microglia at baseline could have potentially been influenced by the increase in both FLI1 expression and phosphorylated AKT at baseline.

Moreover, our study shows that when KO microglia are in a ‘neuroinflammatory’ context or in an ‘AD-context’, they show significant differences in secreted proteins that promote pro-inflammatory activation and mitochondrial metabolism. The pro-inflammatory protein signature in *Trem2* KO, however, was mainly driven by a decrease in IL-10 (suppresses the pro-inflammatory activation of microglia) [49] and an increase in DLK1 (enhances the expression of pro-inflammatory mediators via inhibition of NOTCH1 signaling) [89], compared to *Tyrobp* KO in an LPS context. Additionally, although it has not been directly explored in microglia, the absence of DLK1 has been reported to increase mitochondrial metabolism in other cell types [90], suggesting a dysregulation of mitochondrial metabolism in *Tyrobp* KO microglia. Taken together, these results suggest that pro-inflammatory responses and mitochondrial metabolism are intrinsically different between *Trem2* KO and *Tyrobp* KO microglia.

Some limitations of our study include the analysis of *ex-vivo* microglia, and the use of treatments that mimic ‘neuroinflammation-like’ and ‘AD-like’ contexts *in vitro*. Differences between cultured postnatal microglia and acutely isolated adult microglia have been well documented in prior studies demonstrating that *in vitro* or neonatal microglia downregulate core homeostatic markers such as *Tmem119*, *P2ry12*, and *Sall1* and acquire a partially activated or de-differentiated phenotype [91, 92]. However, within this same culture environment, the *Trem2* KO and *Tyrobp* KO microglia in our study exhibited an even more pronounced activation profile than the WT controls. Because all genotypes were subjected to identical conditions, these differences are unlikely to reflect culture artifacts and instead point to genuine effects of the Trem2/Tyrobp signaling loss. Moreover, by studying microglia in the absence of other cell types, we were able to isolate microglia-intrinsic effects and directly dissect the mechanistic consequences of losing TREM2 or TYROBP within the cell type of interest. Future studies using acutely isolated adult microglia or in situ models such as organotypic brain slices will be important to corroborate and extend these findings in a physiologically relevant context. Nevertheless, our study reports activation patterns like those previously shown in mice in other contexts [93, 94], remaining a powerful tool to understand microglial regulatory mechanisms under both biological and disease-relevant conditions. In addition, although TYROBP can associate with several activating receptors beyond TREM2, such as SIRPβ1 and TREM1 [95–98], and TREM2 itself can signal through other adaptor molecules, such as DAP10 and Plexin A1 [99–102], the present study focused primarily on the canonical TREM2/TYROBP signaling axis. Nonetheless, these alternative receptor–adaptor interactions could contribute to the distinct responses observed in the KO microglia in this study and merit future investigation, particularly in inflammatory or plaque-proximal states.

Because loss-of-function variants in TREM2 or TYROBP cause NHD, our full KO models are also informative for NHD biology, in addition to AD. In NHD, disruption of TREM2/TYROBP signaling is linked to defective microglial phagocytic functions and a dysregulated inflammatory response [103], features that align with the differences we observe between *Trem2* KO and *Tyrobp* KO microglia at baseline and after LPS or Aβ challenges. Overall, our study has revealed intricate mechanisms that are differentially regulated by TREM2 and TYROBP, and are involved in microglial signaling, phagocytosis, migration, and inflammatory signaling, both in homeostasis and during microglial activation triggered by various stimuli. Even though TREM2 and TYROBP closely associate with each other in order to initiate multiple intracellular signaling cascades, our study has shown that these two proteins are not redundant and play distinct and overlapping roles in regulating downstream signaling and transcriptional pathways, and that the underlying mechanisms they modulate to control microglial function differ.

## Supplementary Information

Below is the link to the electronic supplementary material.

Supplementary file1 (XLSX 41.9 KB)

Supplementary file2 (PDF 5.78 MB)

## Data Availability

All raw bulk and processed RNAseq data generated from primary microglia in this study are publicly available at the Gene Expression Omnibus (GEO) (https://www.ncbi.nlm.nih.gov/geo/) under the accession: GSE316055.

## Abbreviations

Aβ: Amyloid beta
AD: Alzheimer’s disease
DAM: Disease-associated microglia
DEG: Differentially expressed genes
DMEM: Dulbecco’s modified Eagle’s medium
HFIP: 1,1,1,3,3,3-Hexafluoro-2-propanol
IPA: Ingenuity pathway analysis
ITAM: Immuno-tyrosine activation motif
KO: Knock-out
LPS: Lipopolysaccharides
MAPK: Mitogen-activated protein kinase
NPX: Normalized Protein eXpression
OA: Okadaic Acid
PBS: Phosphate buffer solution
PEA: Proximity extension assay
PI3K: Phosphatidylinositol-3 kinase
PLCγ: Phospholipase C gamma
PP1/PP2A: Protein phosphatases 1 and 2A
Syk: Spleen tyrosine kinase
ThT: Thioflavin T
TREM2: Triggering receptor expressed on myeloid cells 2
TLR4: Toll-like receptor 4
TYROBP: TYRO protein tyrosine kinase binding protein
WT: Wild-type
