# Supplementary material for: Differential downstream signaling in microglia lacking Alzheimer’s-related TREM2 or its adaptor TYROBP/DAP12
Source: Mol Neurodegener Adv. 2026 Jan 19;2(1):8. doi: 10.1186/s44477-025-00012-x (PMC12872638; doi:10.1186/s44477-025-00012-x)
Supplement: Supplementary file 2 — Supplementary file2 (PDF 5.78 MB) [file 44477_2025_12_MOESM2_ESM.pdf]

Differential downstream signaling in microglia lacking Alzheimer's-related TREM2 or its adaptor TYROBP/DAP12

Gabriela E. Farias Quipildor, Ramona Belfiore, Khaled Althobaiti, Zahra Najarzadeh, Charles Glabe, Benjamin P. Readhead, Sam Gandy, Stephen R.J. Salton, and Michelle E. Ehrlich

Supplementary Figures

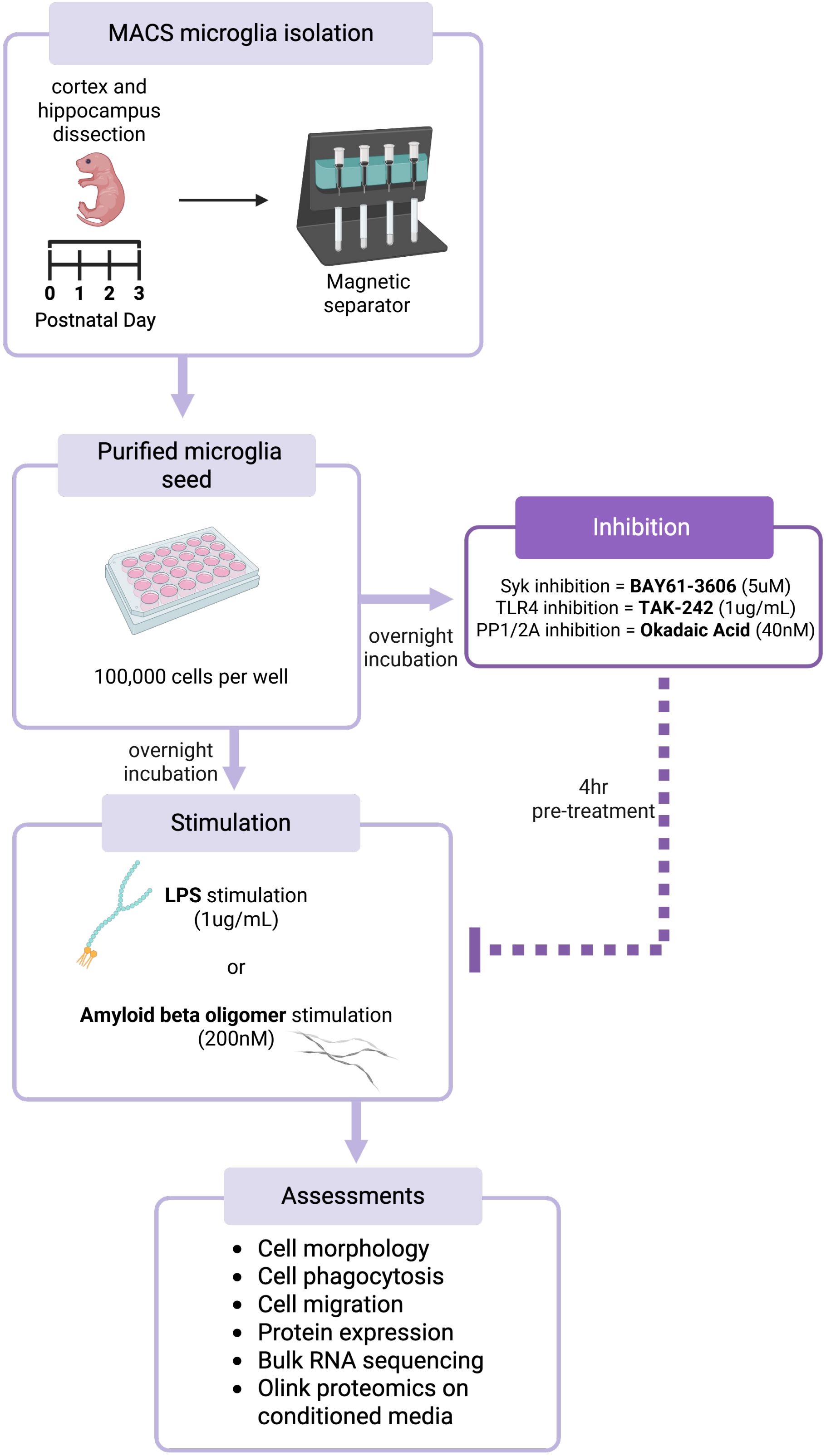
MACS microglia isolation
cortex and hippocampus dissection
0 1 2 3
Postnatal Day
Magnetic separator
Purified microglia seed
100,000 cells per well
overnight incubation
Inhibition
Syk inhibition = **BAY61-3606** (5uM)
TLR4 inhibition = **TAK-242** (1ug/mL)
PP1/2A inhibition = **Okadaic Acid** (40nM)
overnight incubation
4hr pre-treatment
Stimulation
LPS stimulation (1ug/mL)
or
Amyloid beta oligomer stimulation (200nM)
Assessments
Cell morphology
Cell phagocytosis
Cell migration
Protein expression
Bulk RNA sequencing
Olink proteomics on conditioned media

Supplementary Fig. 1 Experimental design summary. Schematic of experimental set-up summary. Microglia were isolated from cortex and hippocampus from postnatal day 0-3 from either WT, *Trem2* KO or *Tyrobp* KO pups using MACS. Purified cells were seeded at a 100,000 cells per well density in 24-well plates and incubated overnight. At this point, primary cells were either pre-incubated for 4 hrs with a Syk, TLR4 or PP1/2A inhibitor (prepared in DMEM unsupplemented media) or DMEM unsupplemented media. After 4 hrs, cells were stimulated either with LPS – for 30 min for western blot signaling analysis or 24 hrs for functional assays, RNA sequencing, and Olink proteomics analysis – or with A11/OC or OC-only preparations – for 24 hrs. Functional assays, protein expression, and RNA transcriptomics were performed on primary cells, and Olink proteomics was performed on conditioned media. This figure was created using Biorender.com.

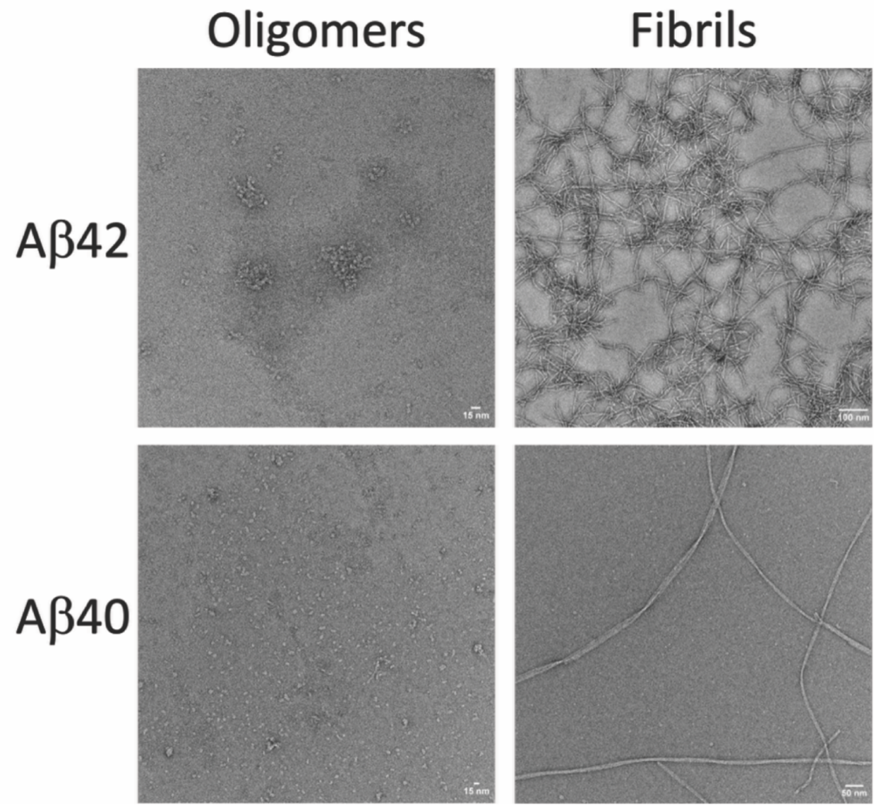

Supplementary Fig. 2 Electron microscopy morphology of the oligomer and fibril preparations. Representative electron microscopy images of Aβ40 and Aβ42, illustrating the morphology of oligomeric and fibrillar preparations.

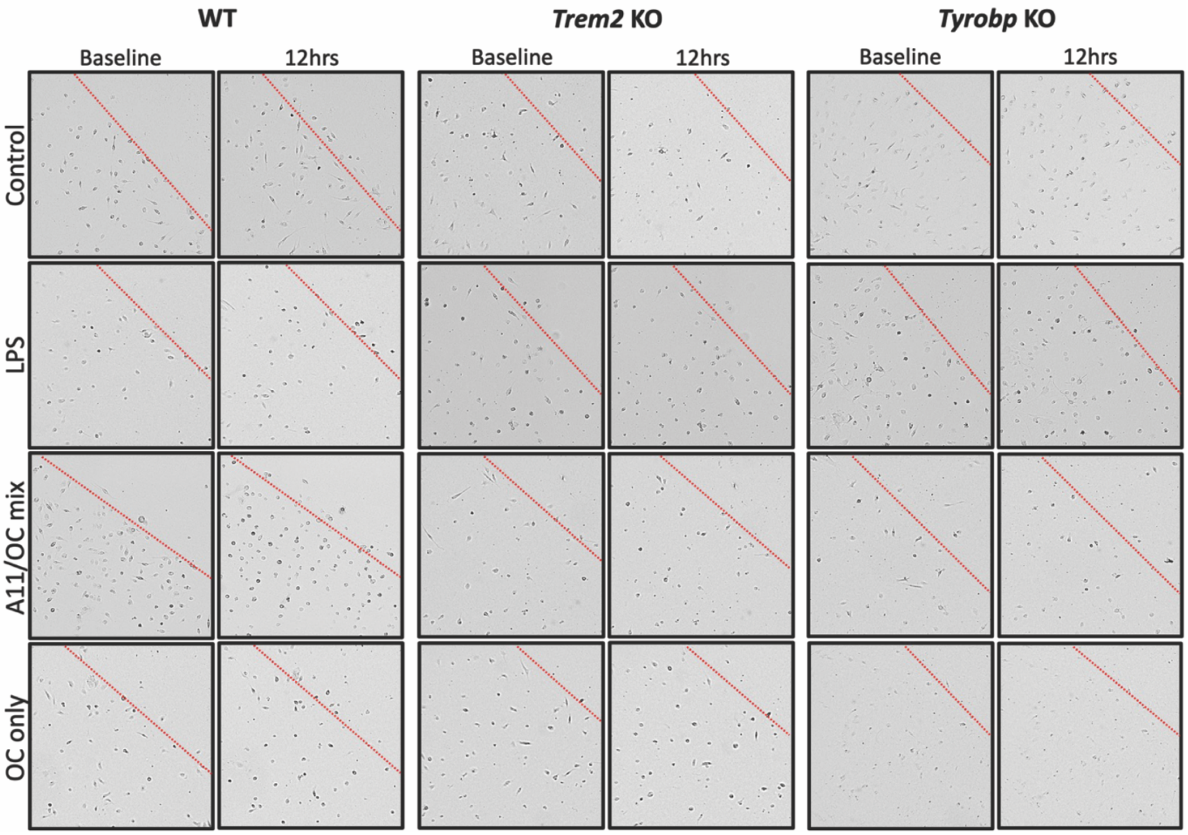

Supplementary Fig. 3 *Trem2* KO and *Tyrobp* KO microglia differ in migration at baseline and after Aβ stimulation. Representative photomicrographs of migrating cells in a scratch-wound assay at 0 and 12 hrs. Images show WT, *Trem2* KO, and *Tyrobp* KO migrating cells at baseline (control in the absence of any stimulation), and after stimulation with either LPS, Aβ pre-fibrillar/fibrillar preparation (A11/OC mix) or Aβ fibrillar preparation (OC-only).

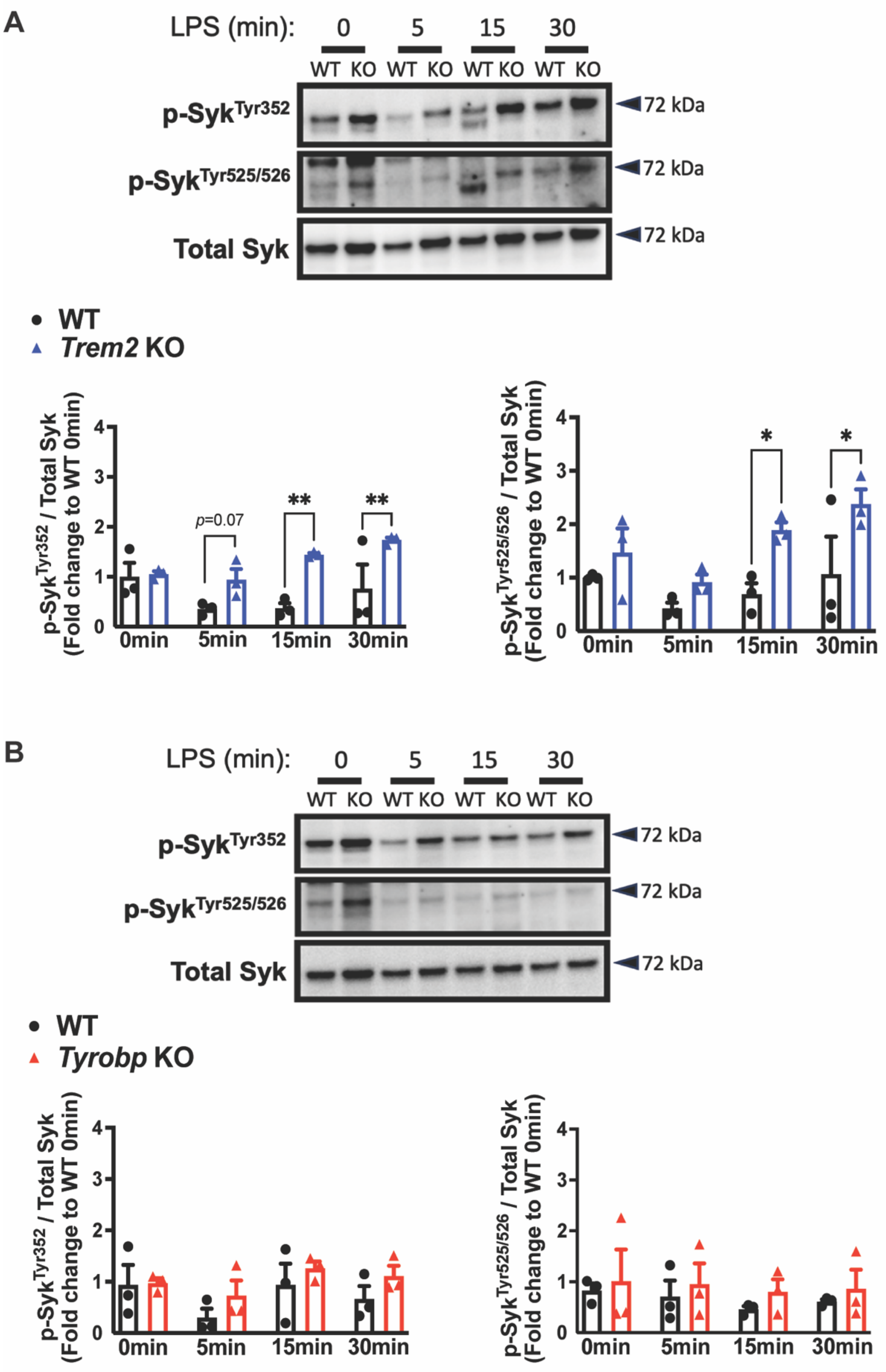

A
LPS (min): 0 5 15 30
WT KO WT KO WT KO WT KO
p-SykTyr352
p-SykTyr525/526
Total Syk
72 kDa
WT
Trem2 KO
p-SykTyr352 / Total Syk (Fold change to WT 0min)
p-SykTyr525/526 / Total Syk (Fold change to WT 0min)
p=0.07
**
*
0min 5min 15min 30min
B
LPS (min): 0 5 15 30
WT KO WT KO WT KO WT KO
p-SykTyr352
p-SykTyr525/526
Total Syk
72 kDa
WT
Tyrobp KO
p-SykTyr352 / Total Syk (Fold change to WT 0min)
p-SykTyr525/526 / Total Syk (Fold change to WT 0min)
0min 5min 15min 30min

Supplementary Fig. 4 Syk signaling did not show differences between KOs and WT controls after rapid LPS stimulation. **A)** Western blot and densitometry quantification for phosphorylated Syk at two sites – p-Syk^{Tyr352} and p-Syk$^{Tyr525/526}$ – and total Syk. Comparison between WT and *Trem2* KO at baseline and after an LPS exposure of 5-, 15-, and 30-min. **B)** Western blot and densitometry quantification for p-Syk^{Tyr352} and p-Syk$^{Tyr525/526}$ and total Syk. Comparison between WT and *Tyrobp* KO at baseline and after an LPS exposure of 5-, 15-, and 30-min. Data was normalized to WT baseline control (WT 0min), and data represented here shows the fold change. *N*=3 independent experiments (data shown here is the average of each experiment). Bars represent means ± SEM. Black bars and individual black-filled circles represent WT microglia, blue bars and individual blue-filled triangles represent *Trem2* KO microglia, and red bars and individual red-filled triangles represent *Tyrobp* KO microglia. Data were analyzed using a two-way ANOVA followed by Tukey's multiple comparisons test. *p<0.05. **p<0.01.

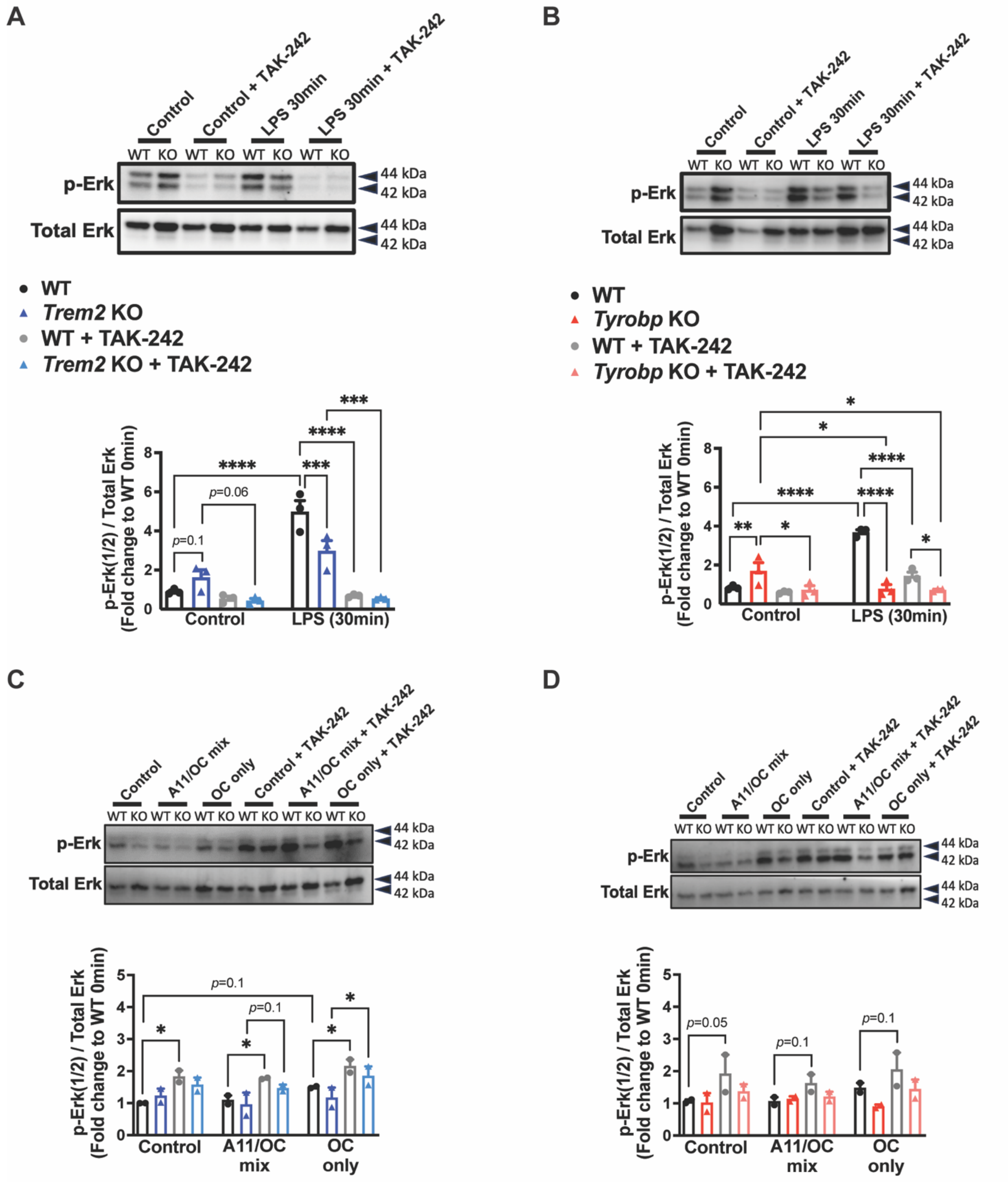
A
Control
Control + TAK-242
LPS 30min
LPS 30min + TAK-242
WT KO WT KO WT KO WT KO
p-Erk
Total Erk
44 kDa
42 kDa
WT
Trem2 KO
WT + TAK-242
Trem2 KO + TAK-242
p-Erk(1/2) / Total Erk (Fold change to WT 0min)
p=0.1
p=0.06
Control
LPS (30min)
B
Tyrobp KO
Tyrobp KO + TAK-242
C
A11/OC mix
OC only
A11/OC mix + TAK-242
OC only + TAK-242
p=0.05
A11/OC mix
OC only

Supplementary Fig. 5 TAK-242 differentially inhibits Erk phosphorylation in KO or WT microglia after rapid or 24-hr stimulation. **A)** Western blot and densitometry quantification for phosphorylated Erk and total Erk after a 30 min exposure to LPS, in the presence or absence of TLR4 inhibitor (TAK-242), for WT vs *Trem2* KO microglia or **B)** WT vs *Tyrobp* KO microglia. **C)** Western blot and densitometry quantification for phosphorylated Erk and total Erk after a 24 hr exposure to A11/OC mix or OC-only, in the presence or absence of TLR4 inhibitor (TAK-242), for WT vs *Trem2* KO microglia or **D)** WT vs *Tyrobp* KO microglia. **A-D)** Data was normalized to WT baseline control, and the data represented here shows the fold change. N=3 independent experiments for the 30 min LPS stimulation tests and N=2 independent experiments for the 24 hr oligomer stimulation tests (data shown here is the average of each experiment). Bars represent means ± SEM. Black bars and black-filled circles represent WT microglia, dark blue bars and dark blue-filled triangles represent *Trem2* KO microglia, and dark red bars and dark red-filled triangles represent *Tyrobp* KO microglia. Gray bars and gray-filled circles represent WT microglia in the presence of inhibitor, light blue bars and light blue-filled triangles represent *Trem2* KO microglia in the presence of inhibitor, and light red bars and light red-filled triangles represent *Tyrobp* KO microglia in the presence of inhibitor. Data were analyzed using a two-way ANOVA followed by Tukey's multiple comparisons test. *$p<0.05$. **$p<0.01$. ***$p<0.001$. ****$p<0.0001$.

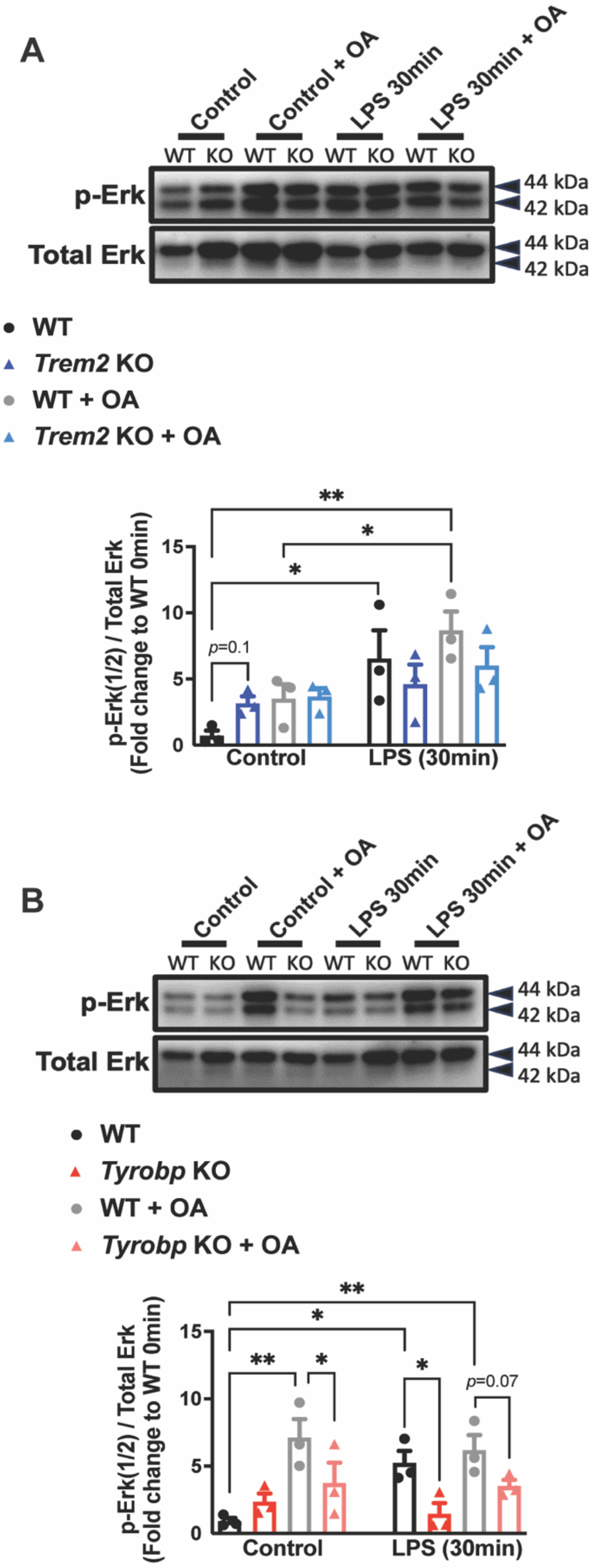

A
Control
Control + OA
LPS 30min
LPS 30min + OA
WT KO WT KO WT KO WT KO
p-Erk
Total Erk
44 kDa
42 kDa
44 kDa
42 kDa
WT
Trem2 KO
WT + OA
Trem2 KO + OA
p-Erk(1/2) / Total Erk
(Fold change to WT 0min)
15
10
5
0
**
*
*
p=0.1
Control
LPS (30min)
B
Control
Control + OA
LPS 30min
LPS 30min + OA
WT KO WT KO WT KO WT KO
p-Erk
Total Erk
44 kDa
42 kDa
44 kDa
42 kDa
WT
Tyrobp KO
WT + OA
Tyrobp KO + OA
p-Erk(1/2) / Total Erk
(Fold change to WT 0min)
15
10
5
0
**
*
**
*
*
p=0.07
Control
LPS (30min)

Supplementary Fig. 6 PP1/PP2A inhibitor shows no difference in ERK activation in LPS-stimulated *Trem2* KO or *Tyrobp* KO microglia. **A)** Western blot and densitometry quantification for phosphorylated ERK and total ERK after a 30 min exposure to LPS, in the presence or absence of a PP1/2A inhibitor (Okadaic acid = OA), for WT vs *Trem2* KO microglia or **B)** WT vs *Tyrobp* KO microglia. **A, B)** Data was normalized to WT baseline control and data represented here show the fold change. *N*=3 independent experiments for the 30 min LPS stimulation tests (data shown here is the average of each experiment). Bars represent means ± SEM. Black bars and black-filled circles represent WT microglia, dark blue bars and dark blue-filled triangles represent *Trem2* KO microglia, and dark red bars and dark red-filled triangles represent *Tyrobp* KO microglia. Gray bars and gray-filled circles represent WT microglia in the presence of inhibitor, light blue bars and light blue-filled triangles represent *Trem2* KO microglia in the presence of inhibitor, and light red bars and light red-filled triangles represent *Tyrobp* KO microglia in the presence of inhibitor. Data were analyzed using a two-way ANOVA followed by Tukey's multiple comparisons test. **p*<0.05. ***p*<0.01.

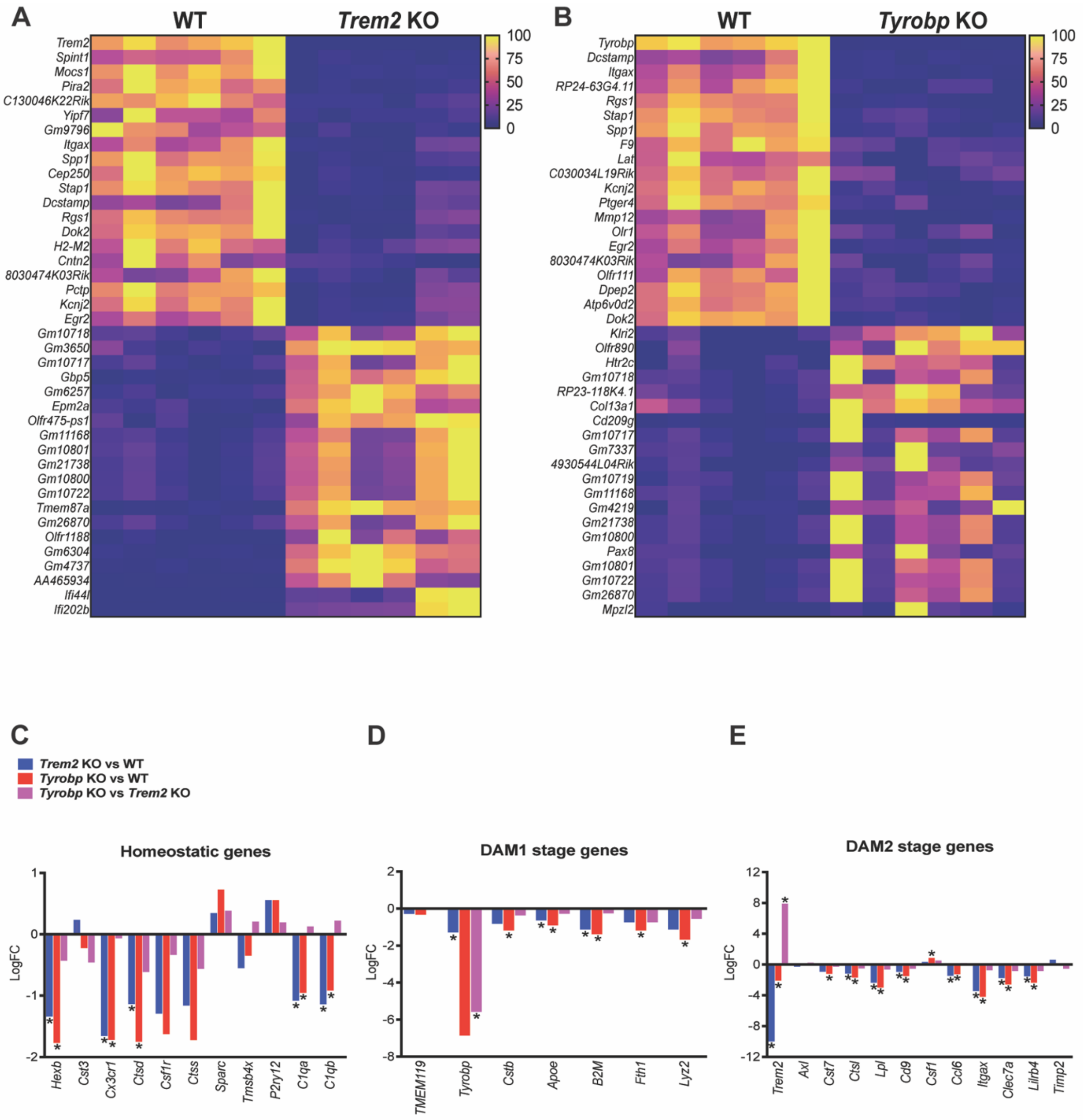

Supplementary Fig. 7 Differential gene expression changes in *Trem2* KO and *Tyrobp* KO microglia at baseline. **A)** Heatmap shows the top 20 upregulated (yellow) and top 20 downregulated (purple) genes for the *Trem2* KO vs WT comparison and **B)** the *Tyrobp* KO vs WT comparison. **C)** Graph of LogFC for microglia homeostatic genes, **D)** stage DAM1 genes, and **E)** stage DAM2 genes. **C-E)** *Trem2* KO vs WT is represented in blue. *Tyrobp* KO vs WT is represented in red. *Tyrobp* KO vs *Trem2* KO is represented in pink. *$p<0.05$.

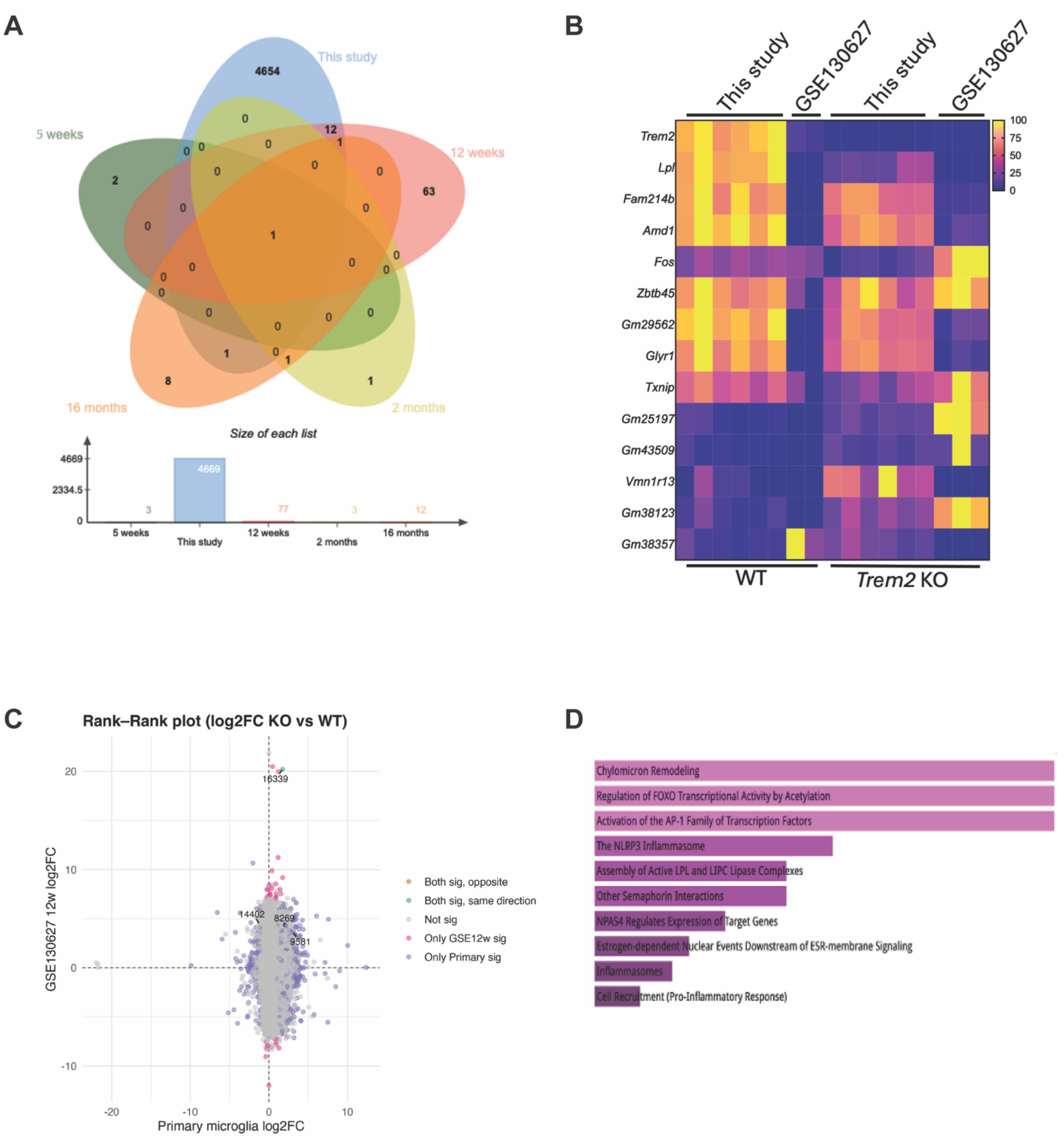
A
This study
5 weeks
12 weeks
16 months
2 months
4654
63
Size of each list
4669
2334.5
5 weeks
This study
12 weeks
2 months
16 months
B
This study
GSE130627
This study
GSE130627
Trem2
Lpl
Fam214b
Amd1
Fos
Zbtb45
Gm29562
Glyr1
Txnip
Gm25197
Gm43509
Vmn1r13
Gm38123
Gm38357
100
75
50
25
0
WT
Trem2 KO
C
Rank–Rank plot (log2FC KO vs WT)
GSE130627 12w log2FC
Primary microglia log2FC
16339
14402
8269
9581
Both sig, opposite
Both sig, same direction
Not sig
Only GSE12w sig
Only Primary sig
D
Chylomicron Remodeling
Regulation of FOXO Transcriptional Activity by Acetylation
Activation of the AP-1 Family of Transcription Factors
The NLRP3 Inflammasome
Assembly of Active LPL and LIPC Lipase Complexes
Other Semaphorin Interactions
NPAS4 Regulates Expression of Target Genes
Estrogen-dependent Nuclear Events Downstream of ESR-membrane Signaling
Inflammasomes
Cell Recruitment (Pro-Inflammatory Response)

Supplementary Fig. 8 Transcriptomic comparison of freshly isolated adult microglia and cultured primary microglia lacking TREM2. **A)** Venn diagram analysis of *Trem2* KO vs WT comparing primary microglia (our study) and microglia freshly isolated at 5 weeks, 12 weeks, 2 months, and 16 months (GSE130627). The graph was created using InteractiVenn [103]. **B)** Heatmap showing the 14 genes that were shared among the datasets, when comparing primary microglia and microglia freshly isolated at 12 weeks (FDR < 0.05). Yellow represents upregulated genes and purple represents downregulated genes. **C)** Rank-rank scatter plot comparing log_2FC values from the microglia acutely isolated at 12 weeks dataset (y-axis) and the primary microglia dataset (x-axis). Each point represents a shared gene after harmonizing identifiers across datasets. Points are color-coded to indicate their significance status. **D)** Reactome pathway enrichment analysis of the significant shared genes among the datasets. Color coding represents enrichment significance (FDR-adjusted p-values), with more intense colors indicating stronger significance.

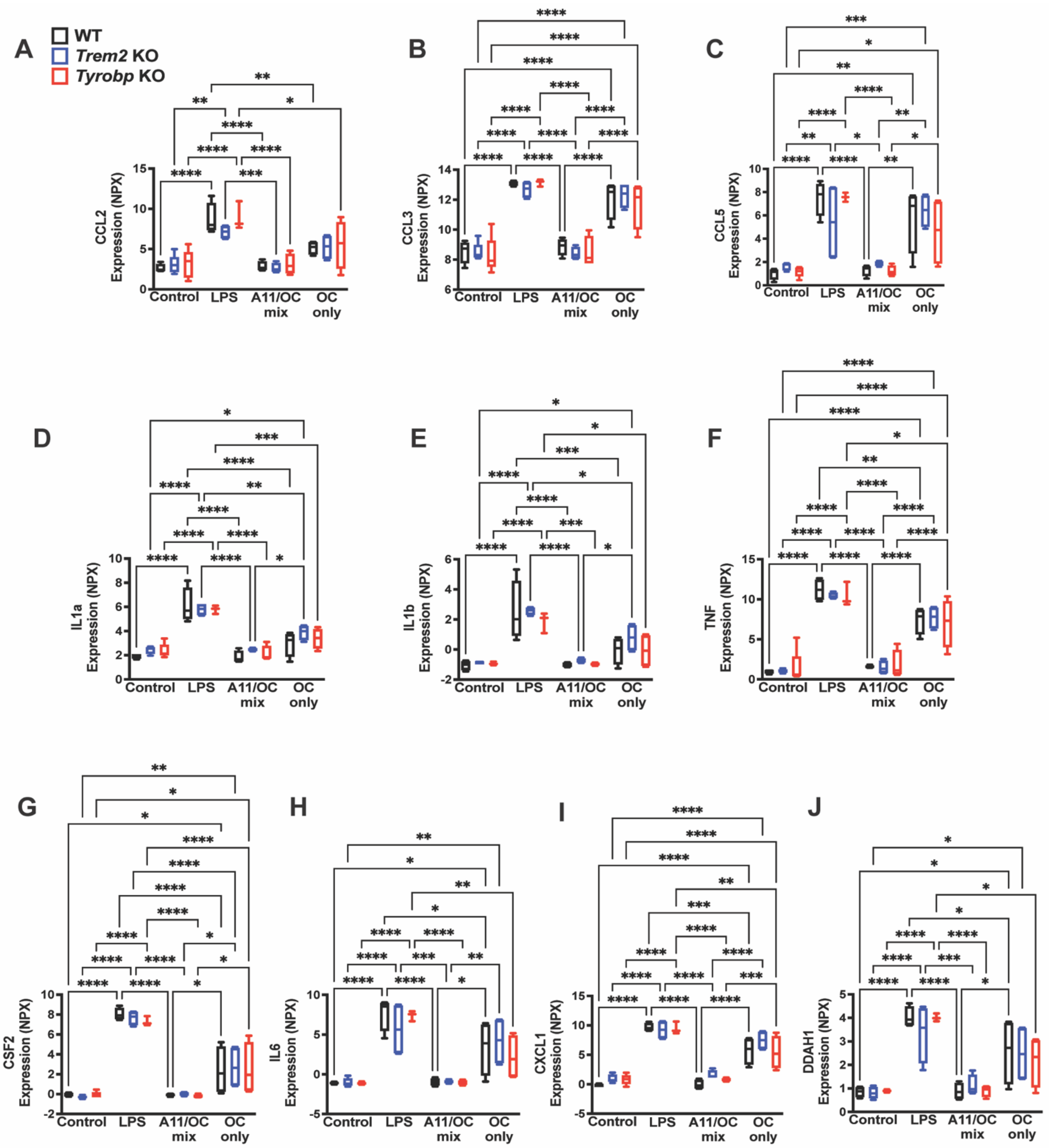

WT
Trem2 KO
Tyrobp KO
A
CCL2
Expression (NPX)
B
CCL3
Expression (NPX)
C
CCL5
Expression (NPX)
D
IL1a
Expression (NPX)
E
IL1b
Expression (NPX)
F
TNF
Expression (NPX)
G
CSF2
Expression (NPX)
H
IL6
Expression (NPX)
I
CXCL1
Expression (NPX)
J
DDAH1
Expression (NPX)
Control
LPS
A11/OC mix
OC only

Supplementary Fig. 9 Proteomic signature in conditioned media from *Trem2* KO and *Tyrobp* KO microglia. **A)** Dot plot showing NPX-values of CCL2, **B)** CCL3, **C)** CCL5, **D)** IL-1A, **E)** IL-1B, **F)** TNF, **G)** CSF2, **H)** IL-6, **I)** CXCL1, and **J)** DDHA1. **A-J)** n=3-5 per genotype per condition. Black bars represent WT microglia, blue bars represent *Trem2* KO microglia, and red bars represent *Tyrobp* KO microglia. Data were analyzed using a two-way ANOVA followed by Tukey's multiple comparisons test. *$p<0.05$. **$p<0.01$. ***$p<0.001$. ****$p<0.0001$.
